# Chemotherapeutic Efficacy of Implantable Antineoplastic-Treatment Protocols in an Optimal Mouse Model for Human Ovarian Carcinoma Cell Targeting

**DOI:** 10.3390/ijms19103030

**Published:** 2018-10-04

**Authors:** Jonathan M. Pantshwa, Khadija Rhoda, Sarah J. Clift, Priyamvada Pradeep, Yahya E. Choonara, Pradeep Kumar, Lisa C. du Toit, Clement Penny, Viness Pillay

**Affiliations:** 1 Wits Advanced Drug Delivery Platform Research Unit, Department of Pharmacy and Pharmacology, Faculty of Health Sciences, School of Therapeutics Sciences, University of the Witwatersrand, Johannesburg, 7 York Road, Parktown 2193, South Africa; monwabisi.pantshwa@wits.ac.za (J.M.P.); dija.rhoda@yahoo.com (K.R.); priyamvada.pradeep@wits.ac.za (P.P.); yahya.choonara@wits.ac.za (Y.E.C.); pradeep.kumar@wits.ac.za (P.K.); lisa.dutoit@wits.ac.za (L.C.d.T.); 2 Department of Paraclinical Sciences, Faculty of Veterinary Science, University of Pretoria, Onderstepoort 0110, South Africa; sarah.clift@up.ac.za; 3 Department of Medical Oncology, Division of Oncology, Faculty of Health Sciences, University of the Witwatersrand, Johannesburg, 7 York Road, Parktown 2193, South Africa; clement.penny@wits.ac.za

**Keywords:** implant, antibody functionalized nanomicelles, epithelial ovarian cancer, chemotherapeutic drugs, nude mouse model

## Abstract

The present study aimed to design and develop a nanocomposite drug delivery system employing an antineoplastic-loaded antibody functionalized nanomicelle encapsulated within a Chitosan–Poly(vinylpyrrolidone)–Poly(*N*-isopropylacrylamide) (C–P–N) hydrogel to form an in situ forming implant (ISFI), responsive to temperature and pH for cancer cell-targeting following intraperitoneal implantation. The optimum nanomicelle formulation was surface-functionalized with anti-MUC 16 (antibody) for the targeted delivery of methotrexate to human ovarian carcinoma (NIH:OVCAR-5) cells in Athymic nude mice that expressed MUC16, as a preferential form of intraperitoneal ovarian cancer (OC) chemotherapy. The cross-linked interpenetrating C–P–N hydrogel was synthesized for the preparation of an in situ-forming implant (ISFI). Subsequently, the ISFI was fabricated by encapsulating a nanocomposite comprising of anti-MUC16 (antibody) functionalized methotrexate (MTX)-loaded poly(*N*-isopropylacrylamide)-b-poly(aspartic acid) (PNIPAAm-b-PASP) nanomicelles (AF(MTX)NM’s) within the cross-linked C–P–N hydrogel. This strategy enabled specificity and increased the residence time of the nanomicelles at tumor sites over a period exceeding one month, enhancing uptake of drugs and preventing recurrence and chemo-resistance. Chemotherapeutic efficacy was tested on the optimal ovarian tumor-bearing Athymic nude mouse model and the results demonstrated tumor regression including reduction in mouse weight and tumor size, as well as a significant (*p* < 0.05) reduction in mucin 16 levels in plasma and ascitic fluid, and improved survival of mice after treatment with the experimental anti-MUC16/CA125 antibody-bound nanotherapeutic implant drug delivery system (ISFI) (*p* < 0.05). The study also concluded that ISFI could potentially be considered an important immuno-chemotherapeutic agent that could be employed in human clinical trials of advanced, and/or recurring, metastatic epithelial ovarian cancer (EOC). The development of this ISFI may circumvent the treatment flaws experienced with conventional systemic therapies, effectively manage recurrent disease and ultimately prolong disease-free intervals in ovarian cancer patients.

## 1. Introduction

Ovarian cancer is one of the fatal female diseases in which many patients are diagnosed at an advanced stage when the disease has already spread beyond the ovaries to the abdominal cavity [1,2,3]. Targeted treatment using biomarkers for OC holds great promise to improve the survival rate of patients with OC. Actively targeted chemotherapy has been a considerable advancement compared to passive-targeting based on the Enhanced Permeability and Retention (EPR) effect. To this end, actively targeted chemotherapeutic drug delivery employing nanotechnology has had a remarkable impact on cancer treatment with the following advantages: (1) Ability to deliver antineoplastic drugs to treat specific cancer metastatic sites; (2) potential reduction in the quantity of drug necessary to achieve a required therapeutic dose at the target cancer tissue; and (3) reduction in the quantity of drug delivered to healthy cells thus decreasing cell cytotoxicity [4,5,6]. In this context, the coating of nanomicelles with specific antibody targeting ligands can assist in conjugation of the nanomicelles to precise biomarkers and antigen receptors over-expressed on OC cells to target tumors with improved accuracy [7,8]. The flexibility of coated amphiphilic nanomicelles is amenable to modified forms of chemotherapy. Targeting cancer cells at a molecular level using nanocarriers such as nanomicelles may be the solution for more personalized chemotherapy. Antibodies are the most significant target ligands that have offered a broad-spectrum of possibilities in terms of drug targeting and accuracy of interaction. Antibody-targeted nanomicelles (or immunomicelles) can be engineered by composite conjugation of antibodies (or nanobodies) onto the trigger (exposed free terminal) of a hydrophilic segment of the nanomicelle. This ensures active targeting nanomicelle preparation without any steric-hindrance for the ligand-antibody [9,10].

Therefore, this study has innovatively focused on the use of mucins (specifically MUC16) for the advanced design of a novel intraperitoneally administered (as opposed to conventional intravenous chemotherapy) OC cell-targeting nanocomposite drug delivery system to significantly improve the chemotherapy of OC. The uniqueness of the delivery system is via the use of antibody-functionalized nanomicelles to specifically target mucin antigens known to be over-expressed in OC cells. To our knowledge, this has not been investigated before. MUC16, specifically, is an eminent cell surface antigen in OC that is shed into the serum and therefore it is also widely used clinically for the diagnosis and management of epithelial OC [11,12,13].

The monoclonal antibody OC125/Mab anti-MUC16 identifies this tumor-associated antigen, which is over-expressed in epithelial ovarian cancer cells, but not in the epithelium of normal fetal and mature ovaries [14]. The non-specific delivery of chemotherapeutics to healthy tissues other than the OC tumors is one of the leading cytotoxicity challenges of chemotherapy. Therefore, this study also focuses on surmounting this challenge by designing MUC16 antibody-functionalized antineoplastic drug-loaded nanomicelles to specifically target OC cells via the peritoneum. However, targeted nanomicelles have a few limitations regarding the ability to provide sustained drug release. The limitations of these nano-enabled polymeric systems include instability in the circulatory system, rapid degradation, and clearance by the immune system and a lack of controlled sustained drug release [15,16]. To overcome these limitations for clinical applications to be feasible, current research focuses on preparation of nanomicelles encapsulated with hydrogels for solid intra-tumoral release of chemotherapeutic drugs over-prolonged periods of weeks, or even months, thus diminishing the dire need for daily administration of systemic chemotherapy. Hence, prolonged circulation in the bloodstream, in-vivo stability, biodegradability, and polymer-drug compatibility with sufficient retention of the drug within the carrier system are prerequisites to successful design and preparation of an implantable drug targeting delivery system [17,18].

## 2. Results

### 2.1. Mechanism of Synthesis of the Anti-MUC16 MTX-Loaded Nanomicelles

**Figure 1:**
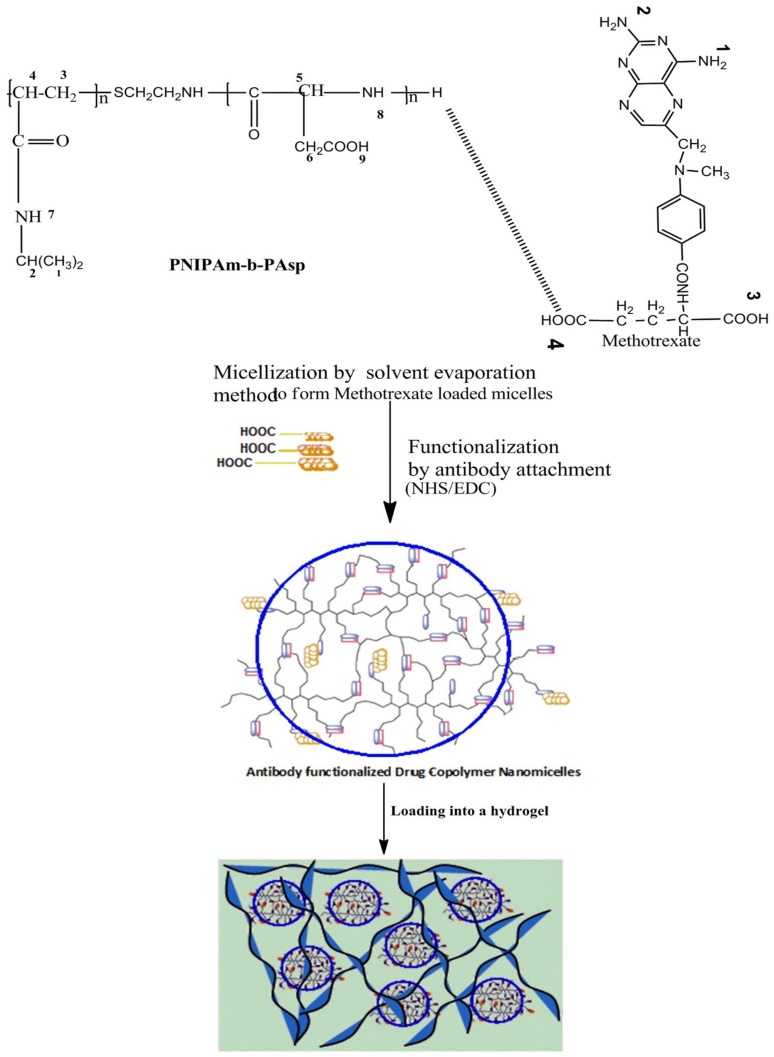
A schematic illustration of the synthesis of an implantable antibody functionalized methotrexate loaded nanomicelles hydrogel composite delivery system (ISFI). Blue circles refer to nanomicelle’s corona.

Amphiphilic block copolymers are expected to offer improved applications in chemotherapeutics when formulated as nanomicelles functioning as nanocarriers for ionic and non-ionic drug loading [19,20,21]. Ionic complexes/composites involving anionic antineoplastic drugs and aspartic acid can be produced and nanomicelles further synthesized by self-assembly of the drug and amphiphilic copolymer in an aqueous medium. In this amphiphilic structure, the hydrophilic surface shields the hydrophobic interior from the interaction of the inner core with the surrounding aqueous environment, thus enabling the nanomicelles to be used as a vehicle for targeted drug delivery (Figure 1).

In this study, a more efficient strategy (under moderate formulation conditions) was utilized to ensure superior targeting of the functionalized core-shell nanomicelles using a simplified synthetic approach outlined in Figure 1. The physical synthesis comprised two synthetic steps: (1) The block copolymer constituted an aspartic (ASP) backbone and poly(*N*-isopropylacrylamide) (PNIPAAm) outer surface to self-assemble the MTX-loaded core-shell nanomicelles by solvent evaporation in an aqueous medium. The polycationic backbone formed between poly(aspartic acid) (PASP) and methotrexate (MTX) generated the inner core of the nanomicelle, while the non-ionic PNIPAAm formed the outer hydrophilic shell. PASP was used to conjugate the MTX (a carboxylic drug) to its amine groups to form an amide (CO-NH) linkage (2). Furthermore, the anti-MUC16 antibody was surface-coated to the nanomicelles via carbodiimide-sulpho-NHS mediated conjugation through carboxylic (-COOH) linkage of the PNIPAAM-b-PASP copolymer. This linkage was also significant for the elucidation of the orientation of the anti-MUC16 to ensure binding affinity and stability. In this case, the MTX-loaded nanomicelles were incubated with 1-ethyl-3-(3-dimethylaminopropyl)-carbodiimide and *N*-hydroxysuccinimide for 15 min at room temperature. The resulting activated nanomicelles were then covalently linked to the anti-MUC16 antibody (1% weight compared with the polymer concentration) (Figure 1). The resultant anti-MUC16 antibody-functionalized MTX-loaded nanomicelles (the NanoComposite) were then lyophilized (Freezone 6, Model 79340, Labconco, MO, USA). 

The results of this study confirmed that MTX can be loaded into the interior of the PNIPAAM-block-PASP nanocomposites and the carboxyl-amine-groups of the nanomicelles were amenable to surface coating with the anti-MUC16 antibody (Figure 1). It is well known that MUC16 is highly expressed on malignant cells and not on healthy cells and therefore the NanoComposite can impede tumor growth and even halt metastasis based on the binding to MUC16. In addition, it is important to note that OC cells also use MUC16 as a mechanism for metastasis via muco-adhesion to other organs within the peritoneum. Thus, the developed NanoComposite has far-reaching potential for the targeted treatment and management of OC employing advanced nanochemotherapeutics.

#### 2.1.1. Analysis of the Molecular Structure Integrity of the Functionalized Nanocomposite

**Figure 2:**
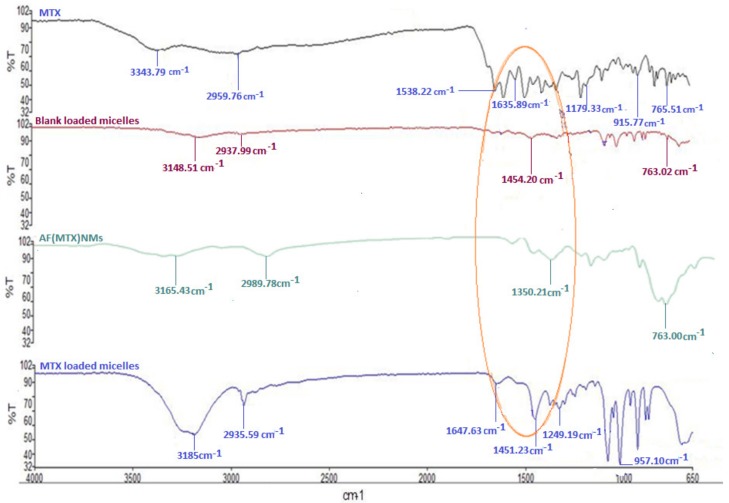
Fourier-transform infrared (FTIR) spectra illustrating the molecular structural transitions of MTX, blank micelles, AF(MTX)NM’s and (MTX)NM’s.

Fourier-transform infrared (FTIR) spectral analysis is one of the most robust analytical techniques to evaluate the molecular structure of block copolymer functional groups [22,23]. The interactions and functionalization of the MTX-loaded nanomicelles with anti-MUC16 was evaluated and described with FTIR spectral analysis and is presented in Figure 2.

As demonstrated, there were distinct chemical structure transitions for the functionalized MTX-loaded nanomicelles compared with the non-functionalized MTX-loaded nanomicelles. The FTIR spectra of the blank nanomicelles and MTX-loaded NanoComposite were congruent with those of the native polymers PNIPAAm and ASP [24,25]. This result showed that the polymer formation into nanomicelles occurred with minor chemical modification during the polymerization process. Hence, the nanomicelles possessed elemental chemical features that were representative of the native polymers. 

Variations were noted in the FTIR spectrum of the blank nanomicelles and MTX-loaded nanomicelles in Figure 2. The additional peaks that were recorded in the MTX-loaded nanomicelles were due to the 1,3-replacement composite (1516.53–1451.23 cm^−1^) and a phenyl amino compound (1647.22–1451.13 cm^−1^). This indicated that MTX was entrapped within the hydrophobic interior of the nanomicelle structure by weak electrostatic H-bonds involving the COO- moiety of MTX and the OH-group of aspartic acid or by the charged ionic groups involving the NH_2_ bonds of MTX and the COO-bonds existing in PNIPAAm-b-PASP.

MTX was dispersed within the PNIPAAm-b-PASP matrix in the amorphous form exclusive of any polymorphic transformation or alteration in the aqueous form. The anti-MUC16 amine groups (N–H) were evident by the 1660.20 cm^−1^ peak as well as the derived amine groups induced by the broad range O–H group and the C–H group observed between 1090.30–1310.11 cm^−1^ that also overlapped the 1000.12–1200.22 cm^−1^ band representative of C–O groups that were attributed to the PNIPAAm-b-PASP copolymer. These transitions indicated that there were prominent interactions between the MTX-loaded nanomicelles and the anti-MUC16 antibody that confirmed the surface coated functionalization of the MTX-loaded NanoComposite.

#### 2.1.2. Analysis of the Thermal Features of the Drug-Free and MTX-Loaded PNIPAAm-b-PASP Nanomicelles

Differential scanning calorimetric investigations were conducted to observe the drug’s physical form in the nanomicelle, since this could affect the drug release from the delivery system. DSC thermograms of MTX, blank micelles and MTX-loaded micelles are depicted in Supplementary Data Figure S1. The DSC curve of the MTX displayed a critical melting peak at 125 °C. MTX-loaded micelles displayed the melting peak for the drug at 145 °C. Incorporation of MTX within the nanomicelle thus elevated the melting point indicative of enhanced thermal stability of the drug. This would suggest that on incorporation of the partially soluble crystalline MTX within the hydrophobic core it was converted to a less crystalline form, resulting in a favorable MTX entrapment efficacy of 80.6 ± 0.3%. The glass transition temperature (*T_g_*) of the co-polymeric nanomicelles is also evident at ~150 °C, correlating with previous findings [25].

### 2.2. Synthesis and Characterization of Chitosan-Poly(N-Vinylpyrrolidone)-Poly(N-Isopropylacrylamide) (C–P–N) Hydrogel

**Figure 3:**
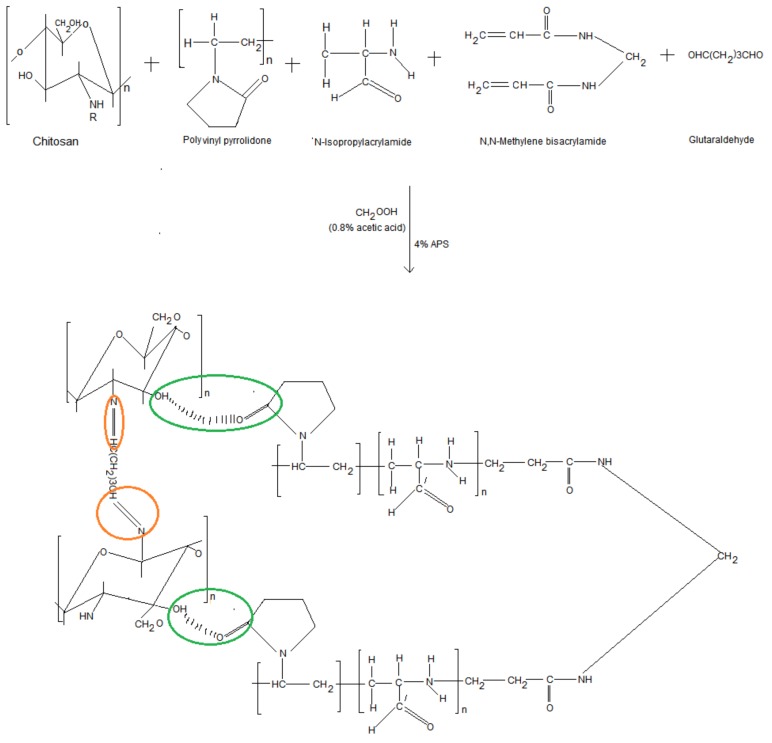
Illustration of constituent chemical structures and reaction mechanism forming composite hydrogel structure.

Figure 3 illustrates the constituent chemical structures and mechanism of synthesis of the C–P–N composite hydrogel structure. Physicochemical characterization via nuclear magnetic resonance (NMR) and FTIR analysis indicated the formation of chitosan-poly(*N*-vinylpyrrolidone)-poly(*N*-isopropylacrylamide) covalent bonds between the protonated amine groups of chitosan (annotated with red circles) and carboxylated groups of poly(*N*-vinylpyrrolidone) (annotated with green circles). Both covalent crosslinking and chemical structure moderated the swelling of the IPN hydrogel. The synthesized C–P–N hydrogel was also verified with chemical structural correlation and proton NMR crest peak assignments (Supplementary Materials Figure S2).

Assessment of Polymeric Structural Variations of the Cross-Linked C–P–N Hydrogel

FTIR analysis of the C–P–N hydrogel proved successful radical polymerization of chitosan (CHT), polyvinylpyrrolidone (PVP) and PNIPAAm, and interpenetration of glutaraldehyde (GA) within the cross-linked network (Supplementary Materials Figure S3). The FTIR spectrum of the C–P–N hydrogel demonstrated peaks at 3428 and 1654 cm^−1^ assigned to stretching vibrations of -NH and -OH, respectively. The peak at 1720 cm^−1^ was attributed to the stretching vibrations of carbonyl group in the PVP-PNIPAAm molecule which differentiated chitosan and the chitosan derivative (CHT-PVP). The PNIPAAm hydrogel sample exhibited significant peaks at 1654, 1551, 1385 and 1369 cm^−1^, which were assigned to the characteristic peaks of amide I, amide II and the isopropyl group, respectively (Supplementary Materials Figure S3). For the composite hydrogel, the intensity of the absorption peak at 1654 cm^−1^ was attributed to increased amide group compared with the CHT-PVP hydrogel due the incorporated NIPAAm. The intensity of the methyl peak and isopropyl peaks decreased when compared with PNIPAAm hydrogel. This indicated successful radical polymerization of CHT-PVP-PNIPAAm and interpenetration by GA and *N*,*N*-methylenebisacrylamide.

### 2.3. Characterization of the In Situ Forming Implant (ISFI)

#### 2.3.1. Assessment of the Gelation Temperature Using Oscillatory Rheology

**Figure 4:**
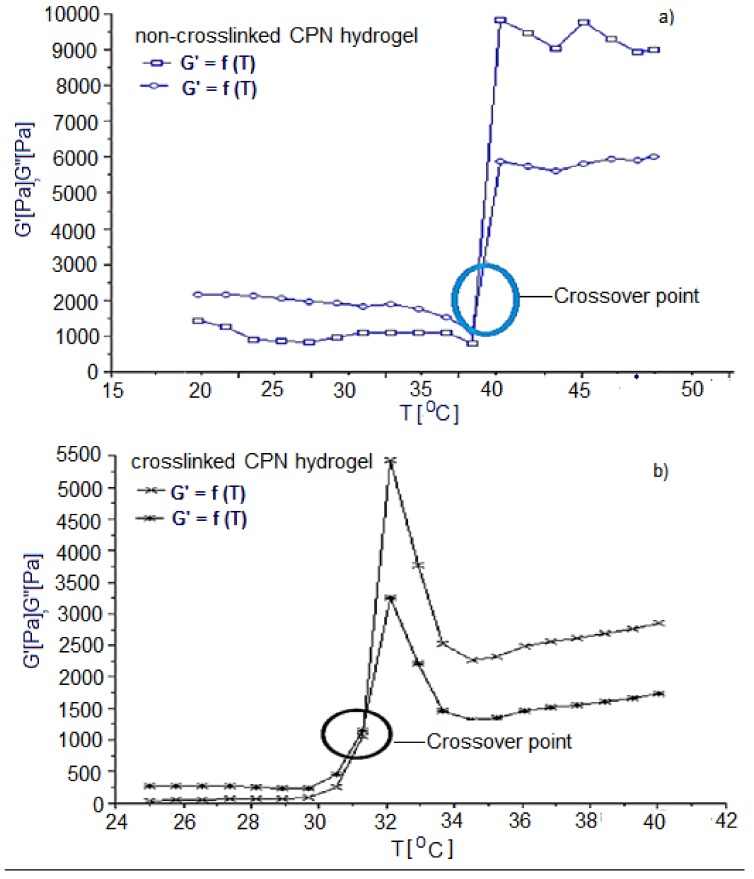
Typical profile obtained during oscillatory rheological testing of non-crosslinked (**a**) and crosslinked (**b**) C–P–N hydrogel samples. The black and blue circles demarcates the crossover point storage modulus (G′) and the loss modulus (G″).

The crossover of the storage and loss modulus (G′ and G″) indicates the gelation temperature (*T_gel_*) of the hydrogel as illustrated in Figure 4. The storage modulus (G′) of a viscoelastic solid is associated with the solid properties or the elastic energy storage properties which indicated that the sample would return to its original state following removal of the deformational energy, while the loss modulus (G″) explains the behavior of the viscoelastic solid when it is acting as a liquid i.e., the viscous properties of the sample representing the dissipation of energy after the application of the deformational stress. Hence at the point at which the storage modulus exceeded the loss modulus, the C–P–N hydrogel was behaving more like a solid than a liquid and hence this was used to determine the thermal gelation temperature. Gelation studies were also conducted on non-crosslinked samples and used as controls (Figure 4).

#### 2.3.2. Morphological Characterization of the ISFI

**Figure 5:**
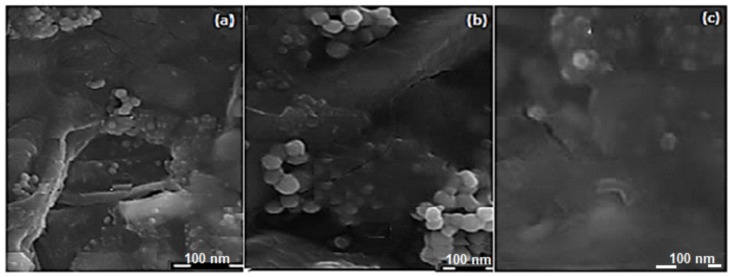
Typical SEM micrographs of the drug-loaded coated nanomicelles after encapsulation in the C–P–N at 50× lower magnification (**a**,**c**); and (**b**) 100× Increased magnification of the ISFI.

The structure and properties of the ISFI were validated by confocal laser scanning microscopy (CLSM) and scanning electron microscopy (SEM). Figure 5 markedly illustrates the morphological properties of the drug-filled coated nanocomposites after encapsulation in C–P–N gel of the ISFI. Drug-loaded coated nanocomposites have a homogenous spherical form. CLSM confirmed optical distribution of the labeled coated nanocomposites in the spatial polymeric-grounded depot platforms. Control native or unlabeled C–P–N hydrogel possessed no rhodamine activities. The overall results substantiate that drug-loaded coated nanocomposites maintain their integrity after-lyophilization for 48 h.

To fully understand the potential of utilizing the ISFI for local drug delivery to human ovarian carcinoma cell, the first factor that had to be assessed was the cytotoxicity. Investigation of in vitro cytotoxicity was conducted on the nanomicelles via MTT assay of OVCAR 5 cells in our previously published work [26]. Similar trends were observed in this study, except that the ISFI enabled a prolonged drug release of 30 days.

### 2.4. In Vivo Studies in the Athymic-Nude Mice Model

In vivo studies were conducted in three platforms: Firstly intraperitoneal (IP) induction of human ovarian carcinoma in Athymic Swiss nude mice, secondly IP implantation of various treatment protocols including ISFI on the optimal ovarian tumor-bearing Athymic nude mouse model, and thirdly in vivo evaluation of chemotherapeutic efficacy utilizing a variety of indices including whole mouse weight, tumor size (measured with calipers and sonography), quantification of mucin 16 antigen expression levels (using immunohistochemistry), and sandwich quantitative CA125 Human ELISA (on plasma and ascitic fluid), as well as the survival rate of mice post-treatment.

#### 2.4.1. Induction of Human Ovarian Carcinoma in Athymic Swiss Nude Mice

Growth of cancer cells in the intra-peritoneal cavity and subcutaneous region of immuno-compromised mice is a common procedure for evaluating tumorigenic potential in vivo [19,20,21]. These procedures are also utilized to evaluate the effects of chemotherapeutic interventions on tumor cell lines. Charles River France Swiss nude mice (*n* = 80), were established as a simple, reproducible mouse model for the induction of human ovarian carcinoma using the IP inoculated NIH:OVCAR5 cell line, as was evidenced by the visible development in vivo of intra-abdominal tumor nodules with associated severe ascites. This occurred within 10 days of inoculation. Advanced ovarian carcinoma disease in the Athymic nude mice was consistently associated with peritoneal carcinomatosis/transcoelomic metastasis, which always preceded the formation of severe ascites. Solid tumor nodules coated all serosal surfaces, especially within the pelvis. These tumor-bearing mice were further IP implanted with various treatment protocols and chemotherapeutic efficacy was evaluated. Due to the widespread dissemination of the IP tumors, it was not feasible to analyze specific regions of the peritoneum to assess the uptake of the antibody (anti-MUC16) and drug-loaded nanomicelle delivery system in the peritoneum.

#### 2.4.2. Chemotherapeutic Efficacy in the Treatment of Human Ovarian Carcinoma

The chemotherapeutic efficacy of the AF(D)NMs implant (the preferred chemotherapeutic model system) was evaluated against the non-functionalized (D)NMs and the comparison group (IV methotrexate/cisplatin only), as well as the control/placebo group. A variety of indices were assessed, including tumor size (measured with calipers and sonography), mouse weight, quantification of mucin 16 antigen expression levels as well as survival rate of mice post-treatment.

#### 2.4.3. Tumor Size

**Figure 6:**
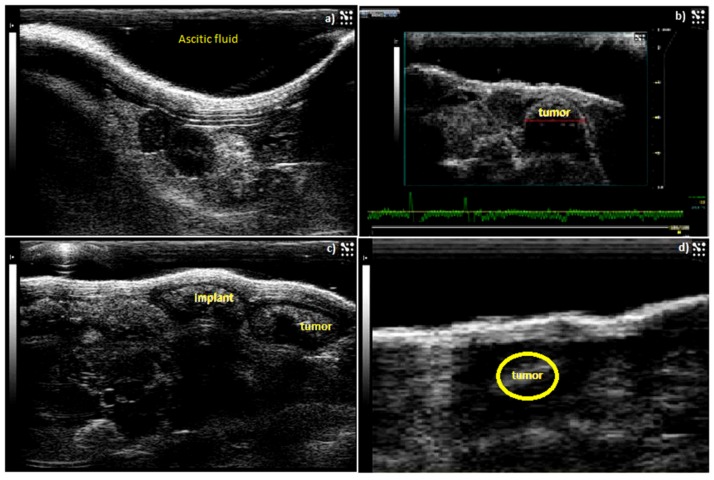
Sonographic representation of IP-tumor growth and response to antibody-bound drug loaded nanomicelle hydrogel composite delivery system (AF(D)NMs). (**a**) Ascitic fluid development in a nude mouse 5 days post-induction; (**b**) Tumor growth 10 days post induction with NIH:OVAR-5 cell suspension; (**c**) Chemotherapeutic implant injected IP-adjacent to tumor growth 11 days post-induction, (**d**) decrease in tumor size and only a small tumor nodule was noticeable 15 days after implementation of the IP-(AF(D)NMs) treatment.

**Figure 7:**
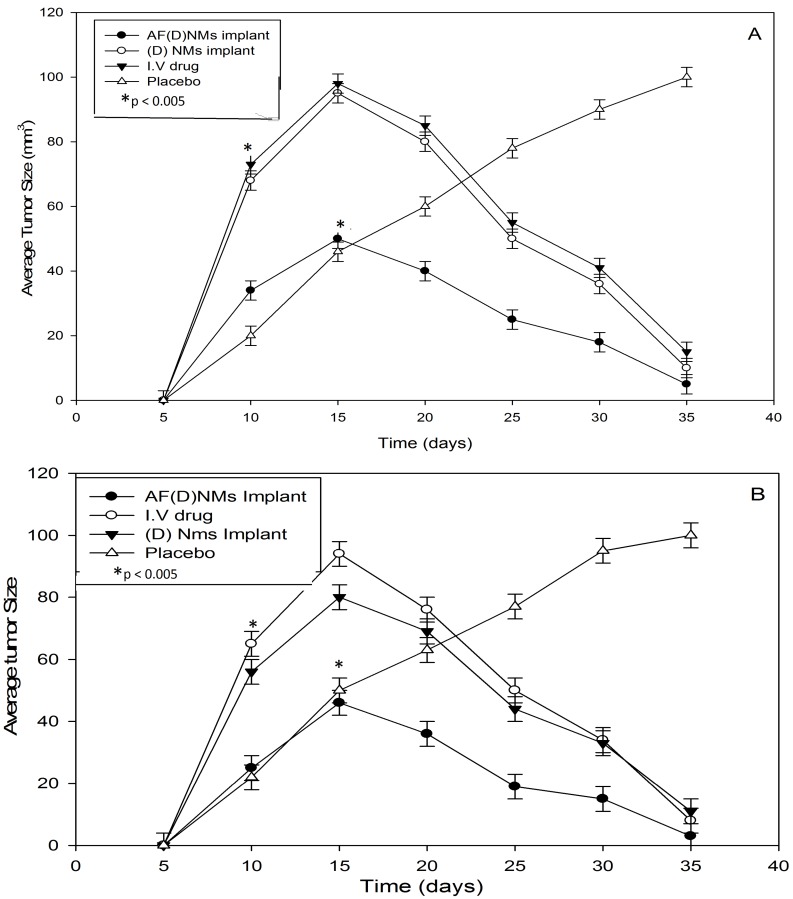
Nude mouse IP-tumor growth curves illustrating chemotherapeutic efficacy of the three treatment groups vs. the control/placebo group, expressed as average tumor sizes in NIH:OVCAR-5 EOC-bearing nude mice. (**A**) refers to the methotrexate and (**B**) the cisplatin-loaded nanomicelle implant delivery system in NIH:OVCAR-5 EOC-bearing nude mice. Each point depicts mean (*n* = 10/group); bar, ±SD. * *p* < 0.005 by *t*-test.

The tumor size was measured with calipers and sonography (Figure 6) and two chemotherapeutic drugs utilized in this study were cisplatin (4 mg/kg) and methotrexate (15 mg/kg), as illustrated with nude mice IP-growth curves, respectively (Figure 7A,B). The average tumor size in the 3 treatment groups each with 10 mice (two experimental groups, and a comparison group (i.v group)) decreased significantly (*p* < 0.05) from day 15 after implantation of the AF(D)NMs treatment. Conversely, in the placebo group, the average tumor size increased steadily, indicating biocompatibility of the blank nanomicelle implant delivery system (placebo) in vivo. During the evaluation phase, nanomicelle implant ((D)NMs)-treated mice reached the ultimate point (of 100 mm^3^ average tumor diameter) within 21 days of treatment whilst the group of AF(D)NMs-implant treated mice survived until the completion of the study. After necropsy examination of the IP inoculated mouse post treatment, the AF(D)NMs implant treatment resulted in reduced average tumor size and ascitic fluid.

#### 2.4.4. Whole Mouse Weight

Mouse weights in both the comparison group (IV cisplatin/methotrexate group) and the 2 experimental treatment groups decreased during this study (*p* < 0.05) (Supplementary Materials Figure S4). Conversely, in the placebo group body weights increased slightly and were normalized to baseline weight (*p* < 0.05). The final average weight of nude mice with NIH:OVCAR-5 tumor treated with AF(D)NMs formulations was 16.94 ± 0.3 g compared with 17.68 ± 0.3 g in mice treated with methotrexate/cisplatin–loaded nanomicelles (D)NMs and 26.34 ± 0.36 g in mice administered with placebo injections (*p* < 0.05). The IV drug group final average weight was 22.62 ± 0.28 g. Baseline weights were 26.00 ± 0.40 g, 24.00 ± 0.33 g and 19.1 ± 0.35 g for placebo, (D)NMs, IV drug and AF(D)NMs, respectively. The body weight of the placebo group was normalized to 26.00 g on day 30, indicative of implant biocompatibility and low levels of cytotoxicity (Supplementary Materials Figure S4).

The notable reduction in tumor size and mouse weights corresponded with decreased MUC16/CA125 antigen expression levels determined with the ELISA technique. These results indicated that there was significant difference (*p* > 0.01) in tumor burden between different chemo-treatment groups and confirmed that antibody functionalized combinational treatment significantly improved chemo-therapeutic efficacy as demonstrated by the inhibition of tumor growth (*p* < 0.05).

#### 2.4.5. Quantification of Plasma and Ascitic Fluid MUC16/CA125 Antigen Levels

**Figure 8:**
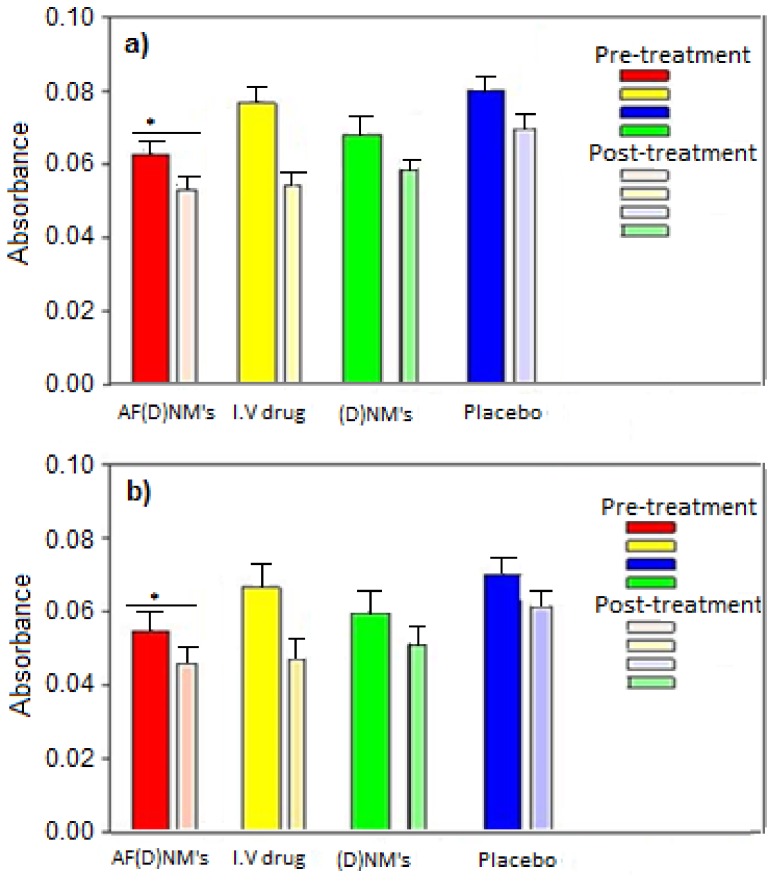
Point-of-euthenasia levels of MUC16/CA125 antigens in the plasma and ascitic fluid of mice in the experimental and control groups. (D) refers to methotrexate in (**a**) and cisplatin in (**b**). Thick and thin bars refer to pre-treatment and post-treatment groups, respectively. *: *p* < 0.05 by *t*-test.

Using the Cancer Antigen CA125 Human ELISA Kit, MUC16 antigen concentrations in IV-treated mouse plasma samples were significantly lower (*p* < 0.05) than the pre-treatment group. However, MUC16 antigen concentrations in mice treated with (D)NMs and AF(D)NMs were all in a lower range, i.e., 1.8–2.4 U/mL. The MUC16 antigen concentration in the plasma samples was, typically, slightly more elevated than in the ascitic fluid, due to the AF(D)NMs site-specificity and localization in the peritoneal cavity. AF(D)NMs target MUC16 antigens expressed on ovarian carcinoma cells within tumor nodules and in ascitic fluid. This AF(D)NMs group significantly reduced MUC16/CA125 antigen concentrations in plasma, as compared to (D)NMs group and IV chemotherapeutic drug group (*p* < 0.05) (Figure 8). At the time of euthanasia, the average plasma MUC16/CA125 concentration in the AF(D)NMs group was 1.9460 U/mL, compared to 2.0180 U/mL in the (D)NMs group, 2.077 U/mL in the IV drug group and 2.368 U/mL in the placebo group. The decrease in MUC16/CA125 antigen concentration in ascitic fluid was consistent with the overall reduction in ascitic fluid production and average tumor size. These results indicate that the AF(D)NMs can specifically target MUC16/CA125 antigens on the surface of EOC cells, thereby effectively decreasing their expression.

#### 2.4.6. IP-Tumor Histopathology

**Figure 9:**
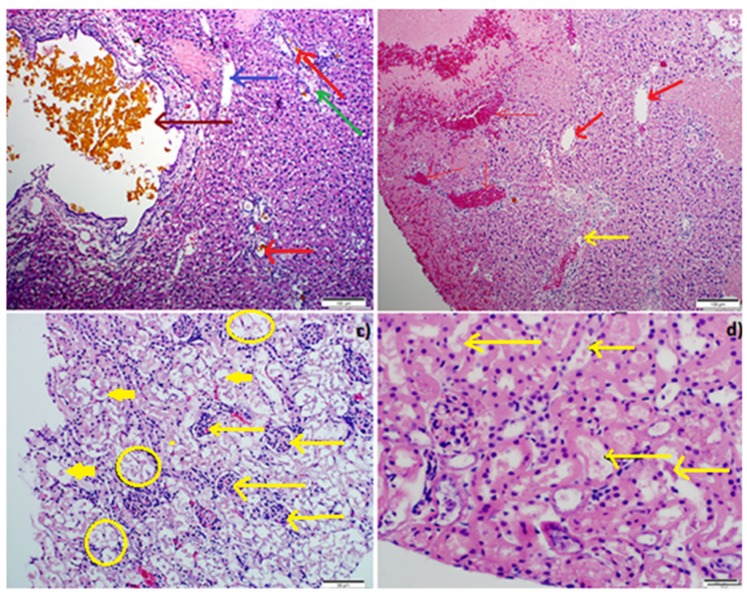
Microscopic view of the liver (**a**,**b**) (scale bar: 100 µm) and kidney (**c**,**d**) (scale bar: 50 µm) in IP-AF(D)NMs, IP-(D)NMs and IV-drug only treatment groups, stained with H and E. (**a**) Cystic distension of bile ductule containing bile (dark brown thick arrows), foci of hepatocellular necrosis (dark blue arrow), mild bile ductule proliferation (red arrows), and mild lymphocytic infiltration into portal areas (green arrow); (**b**) multifocal to coalescing hepatocellular coagulative necrosis with (red thin arrows) or without associated haemorrhage (red thick arrows) which infiltrated with heterophils (yellow arrow); (**c**) severe nephrosis as evidenced by ragged intraluminal clumps of cell debris (yellow rings), inflamed cells (thick short yellow arrows), thin long yellow arrows denote clusters of heterophils HE; and (**d**) basophilic (calcified), granular intra-tubular cellular detritus (yellow arrow), multifocal (segmental) karyolysis in some tubular epithelial cells, and multifocal mild distension of proximal convoluted tubular lumens (lined by slightly attenuated epithelium), HE (Hematoxylin and Eosin staining).

**Figure 10:**
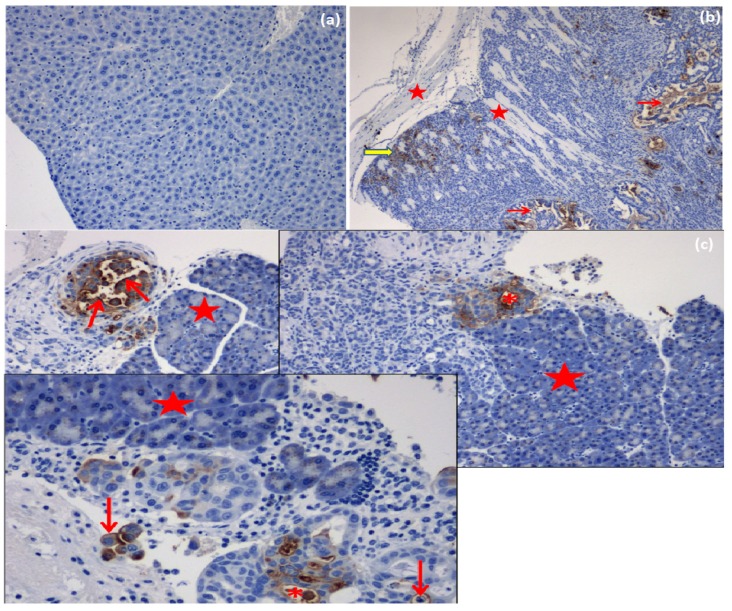
MUC16/CA125-positive labeling of EOC cells in mouse tissues and tumor foci in IP-inoculated nude mice (MUC 16/CA 125, scale bar: 50 µm): (**a**) Negative tissue control (normal mouse liver). MUC16/CA125 immunolabeling, DAB chromogen, hematoxylin counterstain; (**b**) IP-ovarian carcinoma infiltrating skeletal myofibrils (stars) of the body wall. EOC cell-associated (yellow arrow) and extracellular (red arrows) MUC16-positive labeling. MUC16/CA125 immunolabeling, DAB chromogen, hematoxylin counterstain; (**c**) IP-inoculated ovarian carcinoma infiltrating the pancreas (stars) with MUC16-positive labeling of cytoplasmic membranes (arrows) and extracellular (* asterisks) labeling of neoplastic cells. MUC16/CA125 immunolabeling, DAB chromogen, hematoxylin counterstain.

Gross necropsies were performed on all the pre-treatment animals that died or were euthanized by carbon dioxide (CO_2_) inhalation after 10 days. There was no difference in histomorphological appearance of IP-inoculated tumors. Histopathology was subsequently performed in all IP nodules and in all cases, the existence of anaplastic ovarian carcinoma(s) was confirmed (Figure 9). The intra-abdominal tumors, however, were far more infiltrative (widespread transcoelomic metastases with extensive regions of necrosis and histopathology also revealed tumor emboli within lymphatic vessels). Necrosis was linked with a diffuse inflammatory response, comprising of mononuclear cells (Figure 10b). The inflammatory process on the tumor site is controlled by the neutrophil-infiltration. Macrophages are activated to release TNF-α by neutrophil chemotactic factors and reactive oxygen species resulting in the elimination of tumor cells, however an uncontrolled pattern can promote cell laceration and necrosis. After a month of chemotherapy, the mice in the post-treatment groups were euthanized in the same manner as those in the pretreatment group and full liver histopathology performed.

#### 2.4.7. Liver Histopathology

In contrast, tissues sections of nude mice of the treated group demonstrated minor necrotic regions and a lower number of IP-tumor cells (Figure 9). The reduced necrosis was consistent with the decrease in IP tumor-growth. In this vein, histopathology performed on IP nude mice implanted with experimental IV and placebo treatments displayed multifocally coalescing neoplastic nodules throughout the peritoneal cavity, as well as multiple random foci of hepatocellular coagulative necrosis associated with bile “lakes” (so called “bile infarcts”) likely due to biliary outflow obstruction by ovarian carcinomas (in several instances, carcinomatous foci were observed immediately abutting extra-hepatic biliary cysts (Figure 9a,b)). There was also evidence of neoplastic emboli in some sections in the placebo group. There was very mild bile ductile proliferation within the liver sections as well as occasional extra-hepatic biliary cysts (lined by hyperplastic epithelium), the latter also embedded within an increased fibrous connective tissue stroma. Numerous bile ductules within the liver were bile-laden and some bile ductules were severely distended in portal areas and lined by hyperplastic (in certain regions, pseudoepitheliomatous) epithelium. There was a mild portal peri-ductular infiltration of mature (small) lymphocytes and neutrophils and there was evidence of a mild to moderate multifocal portal fibrosis. There was also a venous thrombus in one section of the liver. All these observations are illustrated in Figure 9a,b.

#### 2.4.8. Renal Histopathology

There was widespread but only mild/moderate cortical and medullary intra-tubular cast formation evident as basophilic, granular, and variably vacuolated (derived from degenerate tubular epithelial cells) cellular detritus that filled some tubules. The outer cortex presented with multifocal (patchy) severe nephrosis-lytic necrosis of proximal convoluted tubular epithelial cells, as evidenced by clumps of ragged, mineralized cell debris (due to dystrophic calcification) and occasional karyolitic nuclei in the affected tubular epithelium. In the deeper cortex, there was widespread desquamation/shedding of proximal convoluted tubules (PCT) epithelial cells into tubular lumens, with slight condensation of nuclear chromatin in the affected epithelial cells. There was mild distension of occasional PCT lumens, which were lined by mildly attenuated epithelial cells. All these observations are illustrated in Figure 9c,d.

#### 2.4.9. Immunohistochemistry (IHC)

MUC16/CA125 IHC revealed positive labeling of 1–10% of the area within the specified regions of interest (ROI). Generally, throughout these tumors, variably-sized clusters of neoplastic cells were labeled with the MUC16/CA125 antibody. Labeling was both cellular (cytoplasmic and cell membrane) and extracellular (around shrunken apoptotic-like cells and in tubular lumina lined by irregularly branching papillae of neoplastic epithelial cells). There was occasional distinct membranous labeling of epithelial cells lining the papillary projections in some sections. However, most labeling appeared extracellular in most tumor sections, and there was more cytoplasmic labeling of neoplastic cells compared to membranous labeling (Figure 10a–c).

In addition, the MTX IP delivery by the polymeric ISFI was intracellularly taken up by OVCAR 5 tumor cells via endocytosis. The intracellular-MTX was released by a lysosomal route and distributed to the cytosol where it attached to its dihydrofolate reductase target enzyme and generated a robust S-phase portion. This modification in ovar5 cell cycle provided the anti-proliferative effect in vivo, demonstrated by the regression in tumor volume.

#### 2.4.10. MUC16/CA125 IHC Analysis on Formalin-Fixed, Paraffin-Embedded (FFPE) Epithelial Ovarian Cancer (EOC) Tissue Sections

**Figure 11:**
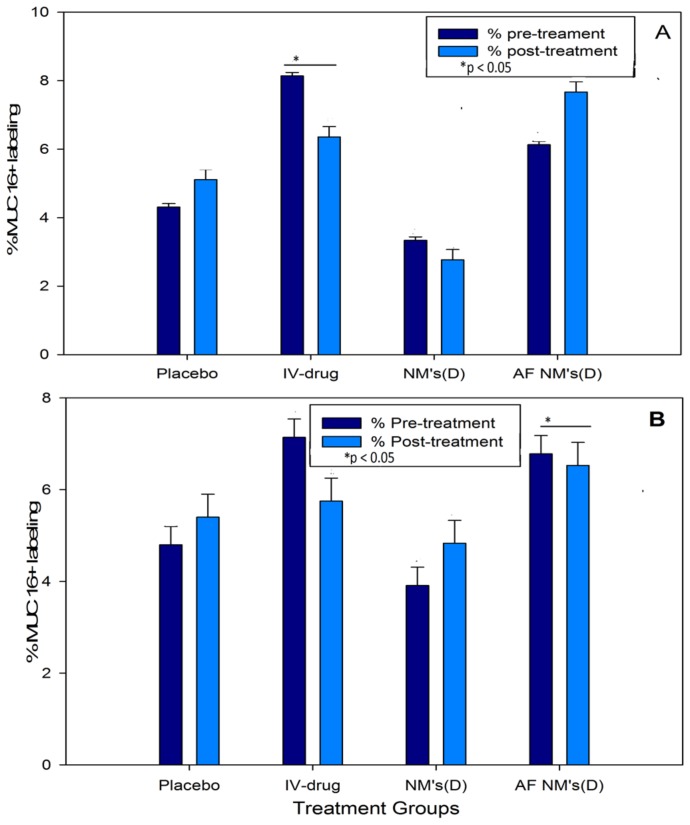
ROI (regions of interest) measurement of IHC images on the stained slides for each treatment group; the MUC16 density in each image was calculated as percentage of MUC16-positive labeling per square centimeter of each selected EOC tissue section (one per mouse). (D) refers to methotrexate (**A**) and cisplatin (**B**). * *p* < 0.05 by *t*-test.

The percentage of MUC16-positive labeling per square centimeter of each selected EOC tissue section (one per mouse) was determined with the aid of the phase separation function for both pre and post-treatment mice. Positive labeling was identified as being brown in color and was observed in both cellular (cytoplasmic and cell membrane) and extracellular locations (in tubular lumina) throughout the neoplastic foci (Figure 11A). MUC16 expression in pre and post-treatment groups using both drugs (methotrexate and cisplatin) was up-regulated and displayed an increasing trend in the placebo (4.31–5.11%), AF(D)NMs (6.13–6.36%) and IV-drug groups (7.67–8.14%) (*p* < 0.05), whilst in the NMs (D) group, MUC16 expression was down-regulated (2.67–3.3%) (Figure 11).

AF(D)NMs reduced CA125 antigens, thereby effectively decreasing their expression. This was validated in quantification of plasma and ascitic fluid MUC16/CA125 antigen levels using the Cancer Antigen CA125 Human ELISA Kit (Figure 8). MUC16/CA125 IHC analysis on FFPE EOC tissue sections (as shown Figure 11B) proved not be a satisfactory tool in differentiation of MUC16 expression levels from pre and post-treatment groups, due to the inaccuracy, ambiguity, and superficial nature of this technique.

**Figure 12:**
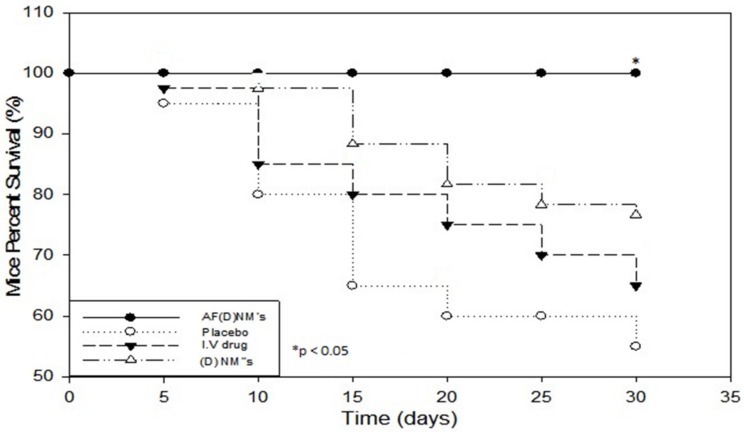
Kaplan-Meier mouse survival curves, showing the three chemo-treatment groups (*n* = 10) including nude mice injected with IP-AF(D)NM’s, IP-(D)NM’s and IV chemotherapeutic drugs and an IP-control placebo implant treatment group. The survival rate of mice in the IP-AF(D)NM’s test group was significantly improved compared with the other groups. * *p* < 0.05.

The survival rate in response to various chemotherapeutic protocols was a significant index for comparing antitumor efficacy between the groups. Nude mice survival rates were significantly different (*p* < 0.05) between the experimental treatment and control/placebo and comparison (IV chemotherapy) groups, as shown by means of Kaplan-Meier analysis. Mouse survival was significantly improved (100% over 35 days) in the AF(D)NMs test group compared to the placebo group, as well as all the other groups (Figure 12). The data also indicated that the mice in the AF(D)NMs test group exhibited the greatest overall reduction in tumor size and had the longest survival times.

#### 2.4.11. Mechanism of Intraperitoneal ISFI Delivery for Human Ovarian Carcinoma Targeting

The ISFI was fabricated by encapsulating a nanocomposite comprising of anti-MUC16 (antibody) functionalized methotrexate (MTX)-loaded PNIPAAm-b-PASP nanomicelles (AF(MTX)NM’s) within a C–P–N hydrogel. Given that the peritoneal cavity is the principal site of disease in ovarian cancer, the ISFI was injected and released nanocomposites into the peritoneal cavity. Following the release of nanocomposites from the hydrogel, the nanomicelles (formulated to circulate for prolonged periods in the peritoneal fluid) targeted specific mucin antigens significantly over-expressed on ovarian cancer cells circulating in the peritoneal fluid (when patients are usually diagnosed) and cancer cells forming nodules at distant sites in the peritoneal cavity. This targeting system reduced the tumor load responsible for adhesion at the sites of secondary metastasis (peritoneal and abdominal surfaces). The anti-MUC16 antibody conjugated nanomicelles has great potential in improvement of tumor selectivity, eliminating/reducing the tumor load, while improving the recovery and long term survival rate of most patients suffering from ovarian cancer.

## 3. Discussion

Ovarian cancer is an insidious female disease that is asymptomatic during its early stages and is usually diagnosed at an advanced, often untreatable stage when the disease has spread beyond the ovaries throughout the abdominal cavity and even further afield [1,2,3]. Thus, deaths due to ovarian cancer could be significantly lowered by developing new, ultrasensitive, yet reliable methods for early diagnosis, and by developing improved treatment protocols. Mucins are among the most promising molecular biomarkers for EOC and have proven invaluable in the diagnosis and monitoring of treatment regimes in various types of ovarian cancer. Increased mucin expression, otherwise known as “mucin switching” that occurs during the neoplastic transformation of ovarian surface epithelium to ovarian carcinoma is important in the progression of this disease [26,27,28]. MUC16 is an important clinical biomarker of ovarian cancer and it is a target for various immuno-chemotherapies currently under investigation [29,30,31].

Blood, serum, plasma, and ascitic fluid levels of MUC16 in mice and humans have been well-researched and are now commonly used as diagnostic markers when evaluating the effect of various chemo-treatments and for establishing the advent of relapse in EOC [32,33]. In this study, quantification of MUC16/CA125 concentration was conducted utilizing the highly sensitive Cancer Antigen 125 (CA125) Human ELISA Kit with a date-to-date coefficient of sensitivity >5%. Progressively increasing MUC16/CA125 values are correlated with ovarian malignancy, whilst steady MUC/CA125 values, even when raised, are correlated with some benign conditions. Significantly, in the pre- vs. post-treatment groups of mice with induced EOC, the MUC16 antigen levels in the blood were 2.368 U/mL in the placebo group, compared to 1.9460 U/mL in the (AF(D)NMs) post-treatment group (Figure 11). Our findings also showed that plasma MUC16 antigen levels were consistent with tumor growth, ascitic fluid development and mouse survival with distinct and combination chemo-treatments, indicative of MUC16 as a valuable biomarker to investigate the effect of chemo-therapeutic interventions on EOC in Athymic Swiss mice.

Histopathology was subsequently performed on all IP nodules and in all cases, the existence of anaplastic ovarian carcinoma(s) was confirmed histologically (Figure 10). Cancer linked inflammation response includes white blood cells infiltration, notably phagocytic macrophage cells, the occurrence of polypeptide representatives of inflammation (as tumor necrosis feature, interleukin-1 and chemotatic chemokines) and the presents of tissue alteration and angiogenesis [34]. This flow of episodes presents an uncontrolled pattern that can promote cell laceration and necrosis. Our findings revealed foci of coagulative necrosis but necrotic foci were most often associated with cholestasis and bile infarcts thought to be due to obstruction to bile outflow by tumor nodules. In prior studies, additional toxic side effects that were observed in association with the use of methotrexate/cisplatin included chronic interstitial, obstructive pulmonary disease, severe renal outer cortex nephrosis [35,36,37]. Immunohistochemical analysis of epithelial ovarian tumors in the present study confirmed an apparent over-expression MUC16 in all induced tumors. Significantly, cellular labeling of MUC16 was detected both in cytoplasmic granular and/or (to a lesser extent) cytoplasmic membrane labeling of neoplastic cells. Our results showed that MUC16 is up-regulated in most of the tumor tissue samples analyzed (Figure 11). As has been shown in previous studies, we also detected a down-regulation of MUC16 expression in ovarian tumors post-treatment as compared to the pretreatment stage. At euthanasia, MUC16 antigen expression in the ovarian carcinoma tissues significantly decreased post-treatment with the AF(D)NMs and only a few small areas of cell debris stained positive in these sections. MUC16/CA125 IHC analysis on FFPE EOC tissue sections (as shown Figure 11B) proved not be an invaluable tool in differentiation of MUC16 expression levels from pre and post-treatment groups, due to the inaccuracy, ambiguity, and superficial nature of this technique. The improved survival rate associated with the AF(D)NMs treatment was probably at least partly due to the profound decrease in ascitic fluid formation in this group (*p* < 0.01). The reduction in ascitic fluid probably occurred due to the specific and prolonged retention of AF(D)NMs hydrogels intra-peritoneally close to the tumor burden; intra-peritoneal tumor cells had close and prolonged exposure to the chemotherapeutic drugs, no doubt resulting in increased drug efficacy in situ. The novel aspect of the study was the design and implementation of an antibody-functionalized nanomicelle hydrogel composite to specifically target MUC16 antigens known to be over-expressed on ovarian cancer cells. Following the release of antibody-bound nanomicelle from the hydrogel, said nanomicelle targeted specific MUC16 antigens over-expressed on ovarian carcinoma cells within the ascitic fluid and within neoplastic nodules at distant sites within the peritoneal cavity. This MUC16 targeting system reduced the mass of neoplastic cells capable of adhering to parietal and visceral peritoneal surfaces at sites distant to the original tumor implant. Chemo-treatment with AF(D)NMs significantly inhibited growth of and transcoelomic metastasis of EOC (via reducing MUC16 antigen expression on tumor xenografts) and reduced the production of ascitic fluid, thereby possessing potential for increasing longevity in patients with ovarian carcinoma.

## 4. Materials and Methods

### 4.1. Materials

Natural and synthetic biopolymers such as Chitosan (MW = 600.000) and Poly(*N*-vinylpyrrolidone) (MW = 40.000) were purchased from Sigma-Aldrich (Steinhelm, Germany). *N*-isopropylacrylamide (MW = 113), poly(*N*-vinylpyrrolidone) (MW = 40.000), *N*,*N*-methylenebisacrylamide, glutaraldehyde (GA) (25%), 2,2′-azoisobutyronitrile, ammonium persulfate, α-amino Aspartic acid, ethanethiol hydrochloride (AET-HCl), 98% analytical grade triphosgene, methotrexate (MTX), 3.5 mg/mL penicillin, 0.1 g/L streptomycin, RPMI 1640 medium, 10% FBS and 0.25% trypsine, and 0.03% EDTA solution were procured from Sigma Aldrich (St. Louis, MO, USA). 98% DMSO, 99.8% anhydrous *N*,*N*′-dimethylformamide (DMF), 99.0 anhydrous tetrahydrofuran (THF), 98.0% diethyl ether, 75% analytical petroleum ether bp (30–60 °C), 1 M hydrochloric acid, potassium chloride (KI), disodium hydrogen phosphate (Na_2_HPO_4_), and potassium dihydrogen phosphate (KH_2_PO_4_) were from Merck Chemicals (Pty) Ltd. (Darmstadt, Germany). NIH:OVCAR-5 cancer cells were procured from director of Fox Chase Cancer Institute, Philadelphia, PA, USA (Dr. Tom C. Hamilton). RayBio^®^ Human CA-125 (MUC16) Elisa Kit for serum, plasma, cell culture media, and urine 96-wells filter plates were procured from Biocom Biotech in (Centurion, Pretoria, South Africa). Anti-MUC16 antibody [OC125] ab693 was procured from Abcam Inc. (Cambridge, MA, USA). Purified deionized water was prepared by a Milli-Q System (Millipore Co., Billerica, MA, USA). Culture plates were purchased from Corning Inc. (Corning, NY, USA). Ovarian cancer cells were cultured in a RS Biotechnological Galaxy (Irvine, UK) incubator kept at 37 °C with 5% carbon dioxide in an entirely humidified environment. Cell experimentations were conducted in the logarithmic stage of development.

### 4.2. Anti-MUC16 Functionalized MTX-Loaded NanoComposite Preparation

#### 4.2.1. Synthesis of PNIPAAm-b-PAS for NanoComposite Formulation

Firstly, a copolymer comprising PNIPAAm-b-PASP was synthesized by solvent evaporation as described previously [38,39]. Thereafter the PNIPAAm-b-PASP copolymer (0.125 g, 0.0079 mmol), anti-MUC16 antibody (0.2 mL, 0.022 mmol), NHS (0.60 mg, 0.0522 mmol), and DCC (10.8 mg, 0.0522 mmol) were dissolved in 10 mL DMF. The solution was mixed under inert conditions at room temperature in the dark for 14 h before dilution with 25 mL deionized water followed by centrifugation to extract DCU. The aqueous supernatant was further extracted by membrane dialysis against deionized water for 24 h with subsequent lyophilization.

#### 4.2.2. Preparation of the MTX-Loaded NanoComposite

MTX-loaded nanomicelles were prepared by solvent evaporation as reported previously [31]. Briefly, 7 mg MTX and 25 mg of PNIPAAm-b-PASP were dissolved in 5 mL DMF and 20 mL distilled water was added drop wise to the solution under homogenization at 360 rpm for 5 min at room temperature. This was followed by solvent removal using a rotary vacuum evaporator to obtain a nanomicelle solution that was then filtrated through a 0.2 μm filter membrane to remove any residual MTX with subsequent lyophilization. The anti-MUC16 antibody functionalized MTX-loaded nanomicelles were prepared with modifications by introducing the anti-MUC16 functionalized block copolymer [40,41]. 

#### 4.2.3. Evaluation of the Molecular Structural Integrity of the Functionalized Nanomicelles

Fourier Transform Infrared (FTIR) spectroscopy (Perkin Elmer Life spectroscopy 100, Lantrisant, Wales, UK) was utilized to differentiate the chemical structure of the block copolymer NanoComposite following nanomicelle coating with the anti-MUC16 antibody, employing a oscillating vibrational component with a diamond interior gauge. Evaluation of Pure MTX, blank nanomicelles, MTX-loaded nanomicelles and the MTX-loaded NanoComposite was conducted at an interval of 650 and 4000 cm^−1^ wavenumber with a 4 cm^−1^ resolution value and smoother 64 scans per spectrum.

#### 4.2.4. Differential Scanning Calorimetry for Elucidation of the Thermal Events of the Methotrexate-Loaded Nanomicelles

Differential Scanning Calorimetry (DSC) studies were conducted with a Mettler Toledo advanced DSC1-STARe equipment from Schwerzenback in Switzerland. The Mettler-STARe software program (version 9, Schwerzenback, Switzerland) was utilized for DSC results acquisition and interpretation. Blank specimen pans were used as reference points and experimental scans were conducted by heating samples at intervals of −10 to 25 degrees for fifteen minutes with a continual isotherm. The lyophilized powder specimens (10 mg) were placed into DSC aluminium-pans and compress-sealed. Relative DSC scans were conducted on blank micelles, MTX-loaded micelles and pure MTX at a heating rate of 10 °C/min from −10 to 325 °C in a stable stream of nitrogen gas. Fresh sample analysis was conducted in triplicate for method development and validation purposes. DSC thermogram patterns were then analyzed for variations in thermal periods. The phase changes of the drug-free micelle and MTX were correlated with the phase changes of the MTX-loaded PNIPAAm-block-PAsp nanocomposites.

### 4.3. Chitosan-PVP-PNIPAAm (C–P–N) Hydrogel Synthesis

Interpenetrating polymer networks (IPN’s) were fabricated by free-radical polymeri-zation mechanism of *N*-isopropylacrylamide distinctive monomers in excipient chitosan and water-soluble poly(*N*-vinylpyrrolidone) (PVP). PVP, *N*-isopropylacrylamide NIPAAm, crosslinkers- *N*,*N*′-methylenebis(acrylamide)-MBAAm, and glutaraldehyde dissolved in aqueous chitosan-CHT (0.02 L, 2%, 1.6% CH_2_COOH-acetic acid), then stirred. CHT, PVP, NIPAAm employed were in 2:2:45 mass percentage. The percentages (*w*/*w* %) between glutaraldehyde-OHC(CH2)_3_HO and CHT were 1%, 2%, 4%. 4% weight percentage existed between MBAAm with NIPAAm *N*,*N*,*N*′,*N*′-Tetramethylethylenediamine (TEMED)/ammonium persulfate (APS) solution was utilized as initiator in the cross-linking process of NIPAAm. Thereafter, APS-TEMED solution (24 μL, 4%) was subsequently added to this reaction mixture. A cross-linked Chitosan**-**PVP-PNIPAAm (C–P–N) hydrogel formed within 1 h of polymerization reaction at 25 °C. After 24 h, the IPN hydrogels were cleaned with deionized H_2_O, then air/vacuum dried. The hydrogel samples prepared were called F_1_ = C–P–N/1, F_2_ = C–P–N/2, and F_3_ = C–P–N/3 IPNs.

#### 4.3.1. Nuclear Magnetic Resonance (NMR) Spectroscopic Analysis 

I^1^H-NMR measurements were applied to the C–P–N components for the confirmation of the copolymer structure and composition. For ^1^H-NMR measurement, 5 mg sample vacuum dried at 50 °C for 48 h was added into a 5 mm NMR test tube, and further vacuum dried at 50 °C for 48 h, to which 500 µL D_2_O solvent was introduced, and lastly the test tube was vortexed to dissolve the polymer in solution. The NMR spectrum was generated using a Bruker DRX400 spectrometer (Bruker, Germany).

#### 4.3.2. Determination of Polymeric Structural Variations

Molecular structural changes in the polymer backbone may alter the inherent chain stability and therefore affect the physicochemical and physicomechanical properties of the selected polymer type for the intended purpose. The molecular structure of native polymers (CHT, PVP, PNIPAAm) the non-cross-linked, and cross-linked C–P–N hydrogel, blank micelles, and drug-loaded micelles were analyzed using FTIR spectroscopy to elucidate any variations in vibrational frequencies and subsequent polymeric structure resulting from drug-co-polymer interaction during nanostructure and hydrogel formation. Samples were analyzed in triplicate at high resolution with scans ranging from 4000 to 400 cm^−1^ on a PerkinElmer Spectrum 100 Series FTIR spectrometer coupled with Spectrum FTIR research grade software (Perkin Elmer Life and Analytical Sciences Inc., Hopkinton, MA, USA).

### 4.4. Preparation of Bio-Responsive IPN Nanomicelle/Hydrogel Composite Based Implant (ISFI)

The ISFI was fabricated by encapsulating antibody functionalized nanocomposite comprising anti-MUC16 (antibody) functionalized methotrexate (MTX)-loaded PNIPAAm-b-PASP nanomicelles within the synthesized bio-responsive C–P–N hydrogel followed by freeze drying. Anti-MUC16 functionalized nanocomposites were encapsulated into the C–P–N at a proportion of 1:5 (NM’s: C–P–N hydrogel). The anti-MUC16 functionalized nanocomposites in solution were introduced drop-wise into the C–P–N gel, and syntheses were permitted to stir until a uniform mixture was reached with a mechanical vortexing system at 37 °C.

#### 4.4.1. Determination of the Gelation Temperature of the Polymeric ISFI Utilizing Oscillatory Rheology

Dynamic rheology is one of the most extensive methods to study rheological properties of polymer hydrogels, and is also the most direct and reliable way for the determination of sol–gel transitions. Viscoelastic properties were measured with a Modular Advanced Rheometer system (ThermoHaake MARS Rheometer, Thermo Fischer Scientific, Karlsuhe, Germany) to indicate the storage modulus (G′), the loss modulus (G″), and tan δ of an aqueous solution of the sol, using cone plate geometry, where the G′ and G″ were recorded under constant deformation. The study of the flow properties was considered extremely significant to this study as it is central to the mechanisms by which the ISFI functions. At room temperature, the implant remains in the liquid state to allow delivery via an 18 G needle and at body temperature (37 °C) the implant forms a solid-like structure. To characterize and analyze the flow behavior and determine the lowest critical solution temperature (LCST) or gelation temperature of the ISFI formulations, rheology studies were conducted using a Haake Modular Advanced Rheometer System (ThermoFisher Scientific, Karlsruhe, Germany). As the polymeric material acts as a visco-elastic solid, some background on viscoelastic solids is provided. To determine the lower critical solution temperature (LCST) and hence the gelation temperature of the ISFI formulation, the temperature of samples was ramped from 20–50 °C at a rate of 0.25 °C/min while applying the predetermined stress of 13.4 Pa, at the frequency of 10 Hz. The gelation temperature was determined as the temperature at which the cross-over of G′ and G″ occurred, i.e., the point at which the formulation was no longer acting as a liquid (G″), but as a solid (G′). In all cases, a solvent trap was used to prevent sample evaporation.

#### 4.4.2. Morphological Characterization of the ISFI

Peripheral morphology, the surface area, and cross sectional area of the ISFI post-lyophilization were observed by using scanning electron microscopy—SEM (JEOL Ltd., JSM-Japanese-Electronic-Optical-Laboratories, Tokyo, Japan). Microscopic observations of the exterior-surface of the nanocomposite configuration of the C–P–N hydrogel was conducted, by first freeze drying the hydrogel at 25 mTorr pressure (Virtis™-Gardiner, New York, NY, USA). The specimen was attached to dual-sided adhesive tape adhered to a metallic specimen stand and sputter-covered with a film of gilded gold. Each specimen was observed under different increasing magnifications at 20 keV-an accelerating-voltage. 

### 4.5. In Vivo Studies in the Swiss Athymic-Nude Mice Model

#### 4.5.1. Mouse Model

Four- to six-week-old female Swiss Athymic nude mice (purchased from Charles River Laboratories International Inc., Ecully, France) were housed in a specific pathogen free (SPF) facility at 25 °C with 60–70% relative humidity (RH) with a 12-h light/dark rotation and then given a week of acclimatization before the experiments commenced. Mice were fed ad libitum diet and water. All procedures were performed under sterile conditions in a laminar flow hood. The animals were monitored daily for general health status. Animal ethics for in vivo studies was approved by University of the Witwatersrand, Animal Ethics Screening Committee (AESC Number: 2012/46/05; Dated 5 December 2012) and the study protocol adhered to these guidelines and those of the South African Council on Animal Care.

#### 4.5.2. In Vitro Cell Culture

The NIH:OVCAR-5 ovarian cancer (OC) cell line is a developed human ovarian epithelial carcinoma cell line that transmits the MUC16 autoantigen [26]. These NIH:OVCAR-5 cells were procured from the director of the Fox Chase Cancer Facility, USA-Jenkintown-CA (T. C. Hamilton). The cells were developed to 80% confluency and attached in plastic culture containers in RPMI growth medium augmented with 10% heat-inactivated FBS, 2 mM glutamine, 50 units/mL penicillin/streptomycin, and 50 units/mL insulin. OC cells were cultured in a RS Biotechnological Galaxy (Irvine, UK) incubator kept at 37 °C with 5% carbon dioxide in an entirely humidified environment. OC cells were weekly sub-cultivated, collected by dissociation of adherent cells with trypsin, twofold cleaned in 10% PBS, dead cells tainted with sterile Trypan Blue and enumerated. Cell culture experiments were performed after 1–2 sub-cultivation rotations. An aqueous 0.4% *w*/*v* trypan blue in sterile 10%, 0.1 M, pH 7.4 PBS was formulated for cell enumeration. A sterile hemocytometer grid was the tool employed for enumeration and a 20 μL cell mass and 60 μL trypan blue volume were collectively mixed. The ratio of live cells was computed as a determinant of the amount of cells enumerated per total number of grid quadrants counted as depicted in % live cell Equation (1). This was 1:3 proportion which generated a 4 dilution-factor.

(1) $$\%\;Live\;cells=\frac{\mathrm{No}\;\mathrm{of}\;\mathrm{cells}\;\mathrm{counted}}{No\;of\;grid\;quadrants\;counted}\;\times DG\times \;10^{4}\;\mathrm{cells}/\mathrm{mL}$$

where *DG* is the dilution-factor utilized and 10^4^ is equation constant. Only specimens that demonstrated viability of more than 95% were used in following analysis.

#### 4.5.3. Induction of Human Ovarian Carcinoma in Swiss Athymic Nude Mice—Pre-Treatment Phase

The ovarian carcinoma induction stage of this study was referred to as the pre-treatment phase. All experiments were conducted inside an Esco Frontier™ DuoFume Hood in the specific pathogen free (SPF) facility. In the present, the model was generated by injecting female Athymic 4- to 6-week-old nude mice IP with 2 × 10^8^ cells/mL of NIH:OVCAR-5 cancer cell suspensions in 0.2 µL RPMI. Inoculations were performed using a 26 G gauge needle and a 1 mL syringe [26]. Human ovarian carcinomas (NIH:OVCAR-5) were induced within 10 days of inoculation. As NIH:OVCAR-5 mainly proliferated within the peritoneal cavity and over-expressed mucin 16 (MUC16/CA125) as compared to normal tissue, it was concluded that the IP growth mimicked human clinical disease. All tumor-bearing mice were further monitored until the tumors developed to their target size of 80–100 mm^3^, which was regarded as the baseline-for initiation of treatment [27]. 

#### 4.5.4. IP-Inoculated Mice

**Figure 13:**
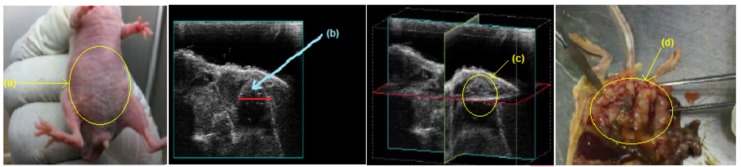
(**a**) An IP-inoculated nude mouse with a moderately distended abdomen due to tumor growth and IP tumor dissemination and ascites; (**b**,**c**) ultrasound images taken using the Vevo^®^ 2100 Imager indicating a tumor diameter of 100 mm^3^ and a 3D image of the circumference of the tumor, respectively; and (**d**) multiple coalescing tumor nodules disseminated throughout the peritoneal cavity (peritoneal carcinomatosis) as seen during the gross necropsy examination.

Mice were inspected weekly and tumor development with peritoneal carcinomatosis in the IP-inoculated mice was monitored based on overall health using the following clinical positive indicators: Presence of intra-abdominal nodular growths, distension of the abdominal cavity due to ascites, weakness, weight loss with extensive skin tenting due to dehydration, and changes in behavior due to perceived pain (Figure 13a). One of the modalities used to assess IP tumor development was a Vevo^®^ 2100 imaging system (Visualsonics Inc., Toronto, ON, Canada), which was used to detect early tumor development (Figure 13b,c). Mice were anaesthetized with Ketamine and Isoflurane before scanning for tumors using the Vevo^®^ 2100 Imager. Tumor-bearing mice were scanned from day 1 PI-post-inoculation (at the site of induction) until day 10 PI and thereafter mice were scanned every second day after implantation of the AF(D)NMs treatment, to evaluate treatment efficacy over the course of therapy (Figure 13b,c).

#### 4.5.5. Experimental Design

Nude mice with growing tumors 10 days post-inoculation were then randomly allocated to five experimental groups including one pre-treatment group (ovarian carcinoma induction group), two experimental groups (a drug loaded nanomicelle ((D)NMs) implant and an anti-MUC16/CA125 functionalized drug loaded nanomicelle (AF(D)NMs) implant group), a comparison group (I.V drug only) and a placebo control group /blank nanomicelle implant group of 10 mice each (*n* = 10/group). Tumor-bearing mice in the pre-treatment group were euthanized by carbon dioxide (CO_2_) inhalation after 10 days once the tumors have developed to their target size of 80–100 mm^3^, which was regarded as the baseline-for initiation of treatment. Organs including the liver, intestines, omentum, mesentery, lungs, pancreas, uterus, oviduct and ovaries, kidney, body wall (skeletal muscle lined by parietal peritoneum), and spleen, as well as solid tumors were collected and ascitic fluid was stored in heparinized tubes (Improve^®^ Improvacuter^®^ Lithium Heparin collection tubes, GmbH, Hamburg, Germany) at −80 °C. The 5 mm^3^ tissue and organ samples were preserved in plastic containers filled 10% neutral buffered formalin for up to 5 days, afterwards the formalin-fixed (FF) samples were submitted to the Section of Pathology (SP), Department of Paraclinical Sciences (DPS), Faculty of Veterinary Science (FVS), University of Pretoria (UP), Onderstepoort (OP) for histopathology, and MUC16/CA125 immunohistochemistry/IHC. The tumor-bearing mice in the 2 experimental, as well as the control and placebo groups, were further subjected to chemotherapeutic efficacy studies. Survival curves were calculated utilizing the Kaplan-Meier method. Survival rate was evaluated as the sum of the days conceded between the introduction of a chemo-therapeutic intervention and mice euthanasia, and percentage (%) nude mouse survival was mice still alive in test groups following introduction of chemo-treatment. 

#### 4.5.6. Chemotherapeutic Efficacy Studies in EOC-Inoculated Nude (NU/NU) Mice

Different chemotherapeutic drugs and formulations were used in each of the two experimental groups/models. The two chemotherapeutic drugs utilized in this study were cisplatin (4 mg/kg) and methotrexate (15 mg/kg). In intervention study 1, anti-mucin 16/CA125antibody-functionalized cisplatin-loaded PEG-PBLG-PF68 nanomicelle implants were utilized, whereas in intervention study 2, anti-mucin 16/CA125antibody-functionalized methotrexate-loaded PNIPAAm-b-PASP nanomicelle implants were used for the targeted treatment of ovarian carcinoma in mice. PEG-PBLG-PF68, PNIPAAm-b-PASP copolymers were utilized in the formulation of the drug-loaded nanomicelle and further encapsulated in hydrogel based implants. These implants were subsequently injected into the mice at a volume of 0.2 mL—(4 mg/kg for cisplatin and 15 mg/kg for Methotrexate) directly into a palpable tumor mass within the peritoneal cavity. Each mouse in the experimental group received a once-off implant treatment monitored for a period of 30 days whilst the comparison group (IV drug only) was administered treatment at 11-day intervals over the period of a month. Mice in all 4 groups were routinely weighed and tumor size was determined sonographically every five days. Mouse masses were consistently correlated with masses at first day of therapeutic administration, to ascertain the ratio mass variation. After a month of treatment, the mice in the post-treatment groups were euthanized in the same manner as those in the pretreatment group. There was no difference in post mortal sample collection post-treatment compared to pre-treatment.

#### 4.5.7. Quantification of MUC16/CA125 Levels in Plasma and Ascitic Fluid

Quantification of the MUC16/CA125 antigen in plasma and ascitic fluid was performed by means of the Cancer Antigen CA125 Human ELISA Kit (Code No. ab108653, Abcam, Cambridge, MA, USA), which is based on the solid-phase assay system. The minimum measurable concentration of mucin 16/CA125 in this Elisa assay was 5 units per mL. For Elisa-analysis, ascitic fluid and whole blood (obtained from cardiac punctures) were collected in heparinized tubes for plasma (Improve^®^ Improvacuter^®^ Lithium Heparin collection tubes, GmbH, Hamburg, Germany). The tubes were centrifuged at 3000 rpm for 5 min to separate the cells from the plasma using a desktop centrifuge (Model TD5A-WS, Shanghai Luxiangyi Centrifuge Instrument Co., Ltd., Shanghai, China). The selected Elisa employs a mouse monoclonal anti-MUC16/CA125 antibody (Mab) against a distinctive antigenic determinant on the intact MUC16/CA125 molecule. The Mab was utilized for solid phase assay immobilization (on the microtiter-wells). Following incubation at 37 °C for 90 min, the microtiter-wells were rinsed with Wash-Buffer to clear unbound-labeled-antibodies. Tetramethylbenzidine (TMB) reagent solution was introduced and incubated for 20 min, resulting in the formation of a blue color. The color-formation was quenched with the introduction of Stop Solution transforming the color to yellow. The concentration of CA125 was directly proportional to the color intensity of the test sample. Absorbance was measured spectrophotometrically at 450 nm.

#### 4.5.8. Histopathology and IHC

Formalin-fixed, paraffin-embedded (FFPE) organs from pre and post-treatment mice (of both cisplatin-and methotrexate-treated nude mouse groups) and IP ovarian carcinomas were sectioned at 3–4 µm and routinely stained with Haematoxylin and Eosin (H&E). Additional 3–4 µm-thick sections were submitted for IHC (specifically the immunoperoxidase labeling technique) to detect membrane-bound and extracellular/shed MUC16. Immunohistochemistry was performed, by hand, following validated protocols. The standard immunoperoxidase procedure for the detection of MUC16 included deparaffinization and hydration of slides, incubation with 3% hydrogen peroxide in methanol for 15 min to quench endogenous peroxidase activity, heat-induced epitope retrieval/HIER (in a microwave using citrate buffer, pH of 6.0 for 14 min at 96 °C), followed by non-specific immunoglobulin binding (Goat blood serum (code nmber G9023 from Sigma Chemical Corporation, St. Louis, MO, USA), PBS diluted to 1:5 factor, pH 7.6, 0.1% BSA for 20 min in 25 °C humidified chamber), incubation for 40 min with the mouse monoclonal anti-MUC16 (Anti-MUC16 antibody [X325] (code no. ab10033, Abcam, Cambridge, UK)) antibody (diluted 1:50 in buffer (0.1 N PBS, pH 7.6, 0.1% BSA)), with subsequent application of the Envision Polymer Detection System (DakoREALEnVision HRP Rabbit/Mouse (ENV) (reference no. K5007, DakoCytomation, Glostrup, Denmark)) according to the manufacturer’s instructions. The reaction product was developed by incubating the tissue sections with a liquid 3,3′-diaminobenzidine (DAB) substrate/chromogen (included in the Envision Immunodetection System) for 1–2 min. Thereafter the portions were counter stainted with Lilly Mayer’s hematoxylin for 20 s, rinsed with water for 10 min, routinely dehydrated through increasing alcohol concentrations and xylol, mounted using Entellan^TM^ (Code No. 1076, Merck Millipore, Darmastadt, Germany) and cover slipped for screening using an Olympus BX43 light microscope. Positive-tissue controls included sections of mouse-inoculated human ovarian carcinoma (NIH:OVCAR-5 cell line) and negative-mouse tissue controls included normal spleen, liver, omental and mesenteric fat, uterus, oviduct, ovary, and pancreas. For negative reagent control purposes, buffer^c^ was substituted for the primary antibody. 

#### 4.5.9. Immunohistochemical Quantification of MUC16/CA125 Antigens in FFPE Tissue Sections

**Figure 14:**
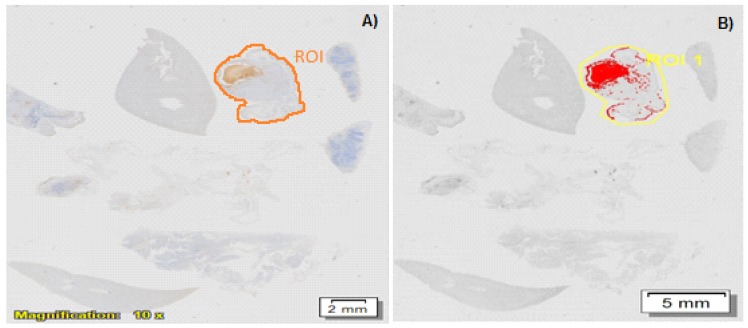
(**A**) Scanned IP-implanted, AF(D)NMs-treated nude mouse tissue section showing a section delineated (brown line) of ovarian carcinoma expressing MUC16/CA125 antigens (brown staining). MUC16/CA125 IHC, DAB, chromogen, hematoxylin counterstain. (**B**) Shows the region of interest/ROI delineated (yellow line) and MUC16-positive signal (red) within the ROI for quantification using the Olympus Count and Measure function (Olympus-Cell Sens software).

All IHC-labeled tissue slides (one slide per mouse) were scanned utilizing the Olympus dotSlide scanner (VS120-S6-W slide loader system in the Department of Anatomical Pathology at the Medical School, University of the Witwatersrand) for the generation of virtual slide images. The single neoplastic nodule with the most MUC16-specific positive labeling (as assessed with the naked eye) per slide was selected for quantification. The dimension count and measure module from the corresponding Olympus-Cell Sens software version 1.12 (Wirsam-Scientific and Precision Equipment-PTY LTD., Johannesburg, South Africa) was utilized to delineate and measure the area of each selected region of interest (ROI) per slide, and the calculation of the percentage IHC positive labeling within each ROI (per mouse) was performed with the assistance of the phase separation function (Figure 14A,B).

#### 4.5.10. Experimental Data Statistical Analysis

Experimental data were evaluated as the mean/average of the data acquired, and the margin of error calculated via the standard-deviation (SD). Statistical analysis was performed by the repeated measures ANOVA. Significant differences between means of treated and untreated groups were analyzed for statistical significance using the two-tail student’s *t*-test for paired/unpaired observations. Two-sided *p*-values < 0.05 were statistically significant. Kaplan-Meier mouse survival curves were prepared and were utilized to ascertain whether MUC16 expression correlated with mouse survival. Mouse Survival rate was computed as the number of days between commencement of the therapeutic regime and mice euthanasia, and mice survival percentage (%) was the total mice existing in each therapeutic group after conception of appropriate therapeutic regime. Kaplan-Meier Survival curves were evaluated, and the variance in mice survival was assessed for statistical significance. Sigma plot 11 graphing software excel (Systat Software, Inc., Richmond, CA, USA) was used for experimental data statistical exploration.

## 5. Conclusions

The present study has demonstrated for the first time that combining anti-MUC16 antibodies with drug-loaded nanomicellles in a hydrogel composite can inhibit intra-peritoneal tumor growth (and therefore peritoneal carcinomatosis), reducing the consequent production of ascites, resulting in increased survival rate of the Athymic Swiss nude mouse model. Our strategy of employing monoclonal antibodies for targeting ovarian cancer cells generated specificity to cancer cells that express MUC16. This functionalized nanomicelle may offer the groundwork for minimizing the cisplatin or methotrexate quantity utilized, thereby decreasing potentially harmful (toxic) side effects without significantly impacting on treatment efficacy. The actively targeted nature of this drug delivery system lends applicability to other solid tumors since targeting modalities that are specific to ovarian cancer have been employed. Hence, potential for use in other solid tumors should be investigated. As a result, this antibody-bound nanotherapeutic implantable drug delivery system may be a potent immuno-chemotherapeutic treatment that can be effectively employed in cases of advanced, and/or recurring, metastatic EOC. In conclusion, in vivo studies showed promising results with the prolonged release of drug from the implant exceeding a month and improved biocompatibility of the ISFI. 

## Supplementary Materials

Supplementary materials can be found at http://www.mdpi.com/1422-0067/19/10/3030/s1.
