# Supplementary material for: Chemotherapeutic Efficacy of Implantable Antineoplastic-Treatment Protocols in an Optimal Mouse Model for Human Ovarian Carcinoma Cell Targeting
Source: Int J Mol Sci. 2018 Oct 4;19(10):3030. doi: 10.3390/ijms19103030 (PMC6213745; doi:10.3390/ijms19103030)
Supplement: Supplementary file 1 [file ijms-19-03030-s001.pdf]

Article

Chemotherapeutic Efficacy of Implantable Antineoplastic-Treatment Protocols in an Optimal Mouse Model for Human Ovarian Carcinoma Cell Targeting

Jonathan M. Pantshwa [1], Khadija Rhoda [1], Sarah J. Clift [2], Priyamvada Pradeep [1], Yahya E. Choonara [1], Pradeep Kumar [1], Lisa C. du Toit [1], Clement Penny [3] and Viness Pillay [1*]

[1] Wits Advanced Drug Delivery Platform Research Unit, Department of Pharmacy and Pharmacology, Faculty of Health Sciences, School of Therapeutics Sciences, University of the Witwatersrand, Johannesburg, 7 York Road, Parktown 2193, South Africa; monwabisi.pantshwa@wits.ac.za (J.M.P.); khadija.rhoda@students.wits.ac.za (K.R.); priyamvada.pradeep@wits.ac.za (P.P.), yahya.choonara@wits.ac.za (Y.E.C.); pradeep.kumar@wits.ac.za (P.K.); lisa.dutoit@wits.ac.za (L.C.d.T.)

[2] Department of Paraclinical Sciences, Faculty of Veterinary Science, University of Pretoria, Onderstepoort, South Africa; sarah.clift@up.ac.za (S.J.C.);

[3] Department of Medical Oncology, Division of Oncology, Faculty of Health Sciences, University of the Witwatersrand, Johannesburg, 7 York Road, Parktown,2193, South Africa; clement.penny@wits.ac.za (C.P.)

*Correspondence: viness.pillay@wits.ac.za; Tel.: +27-11-717-2274

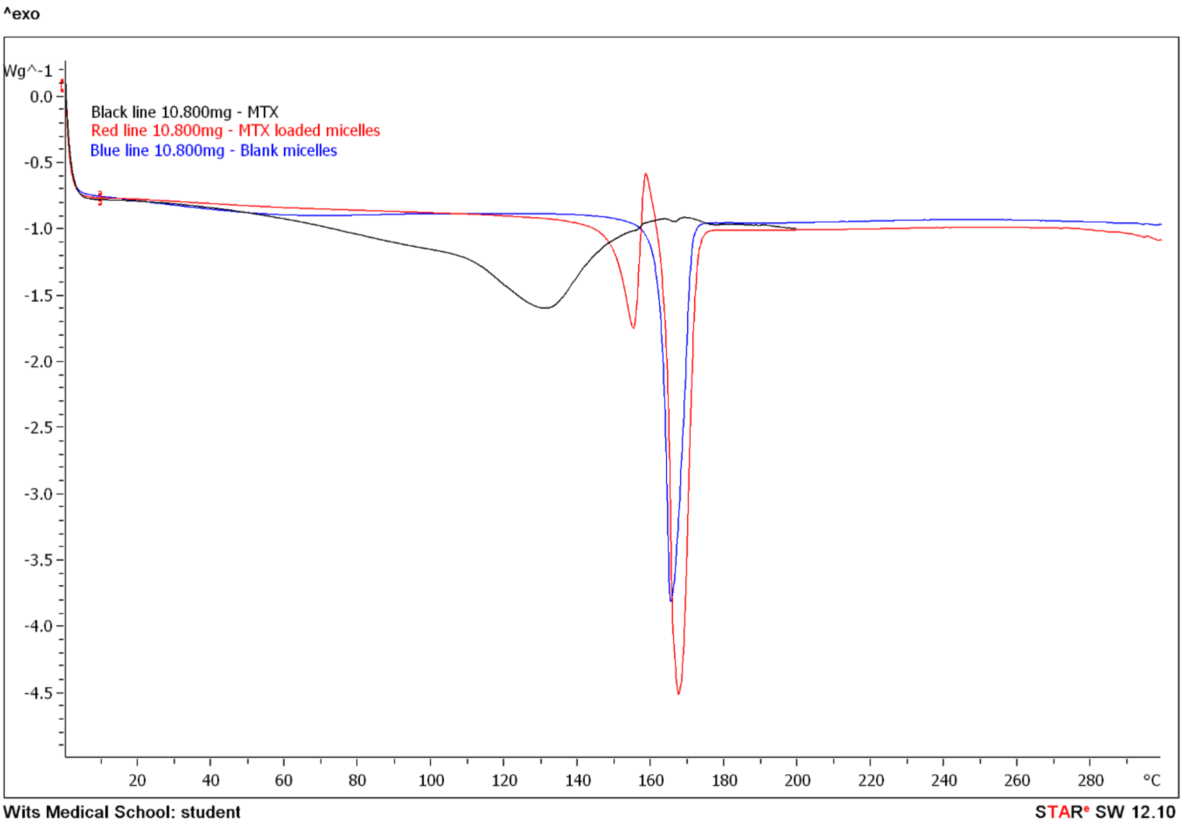

Figure S1. DSC thermograms of pure MTX (black graph), blank micelle nanoformulation (blue graph), MTX-loaded micelle formulation (red graph) demonstrating temperature variations and shifts in the curves.

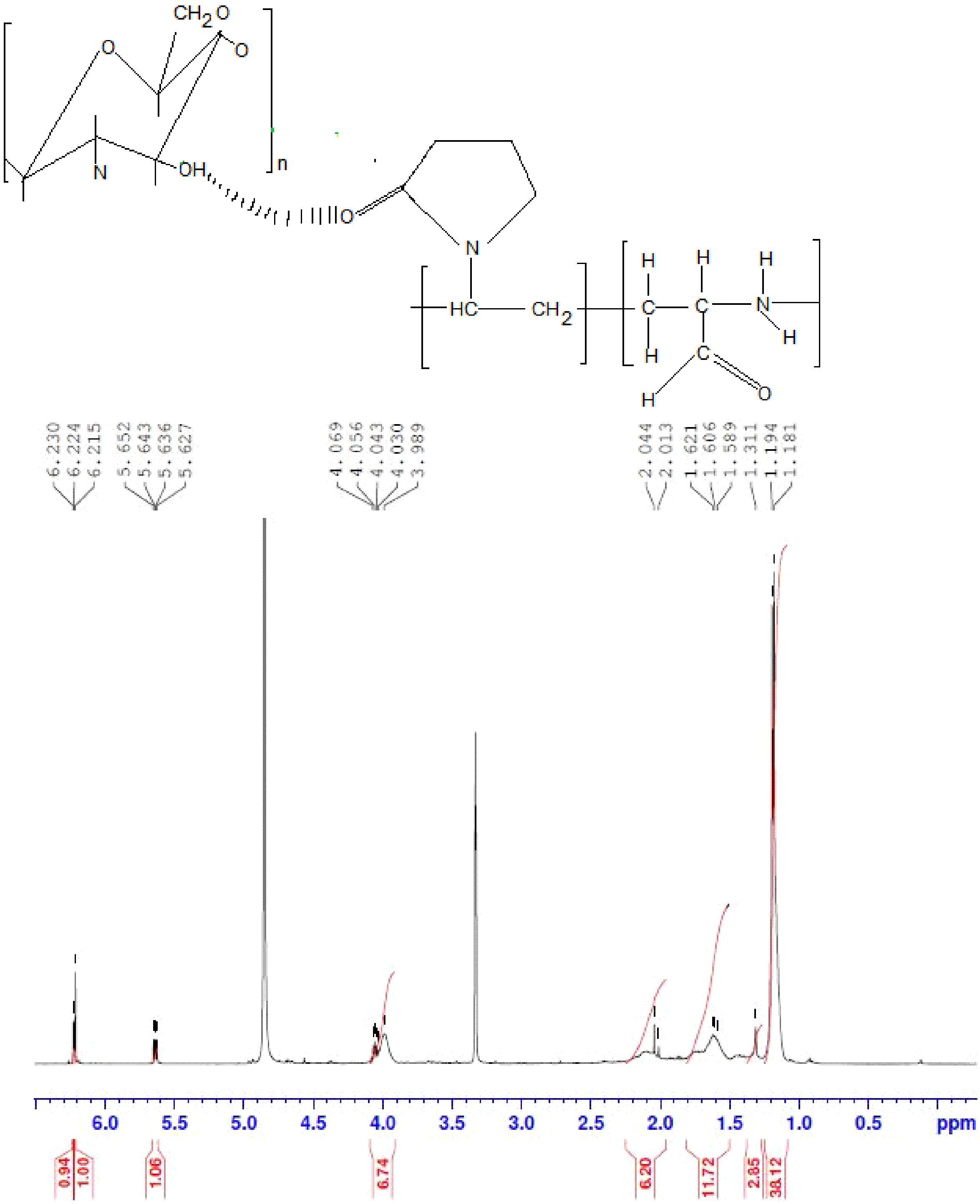

Figure S2: ^{1}H NMR spectra of CHT-PVP-PNIPAAm hydrogel in D_2O and crest peak assignment affirmative to the synthesized composite C-P-N structure.

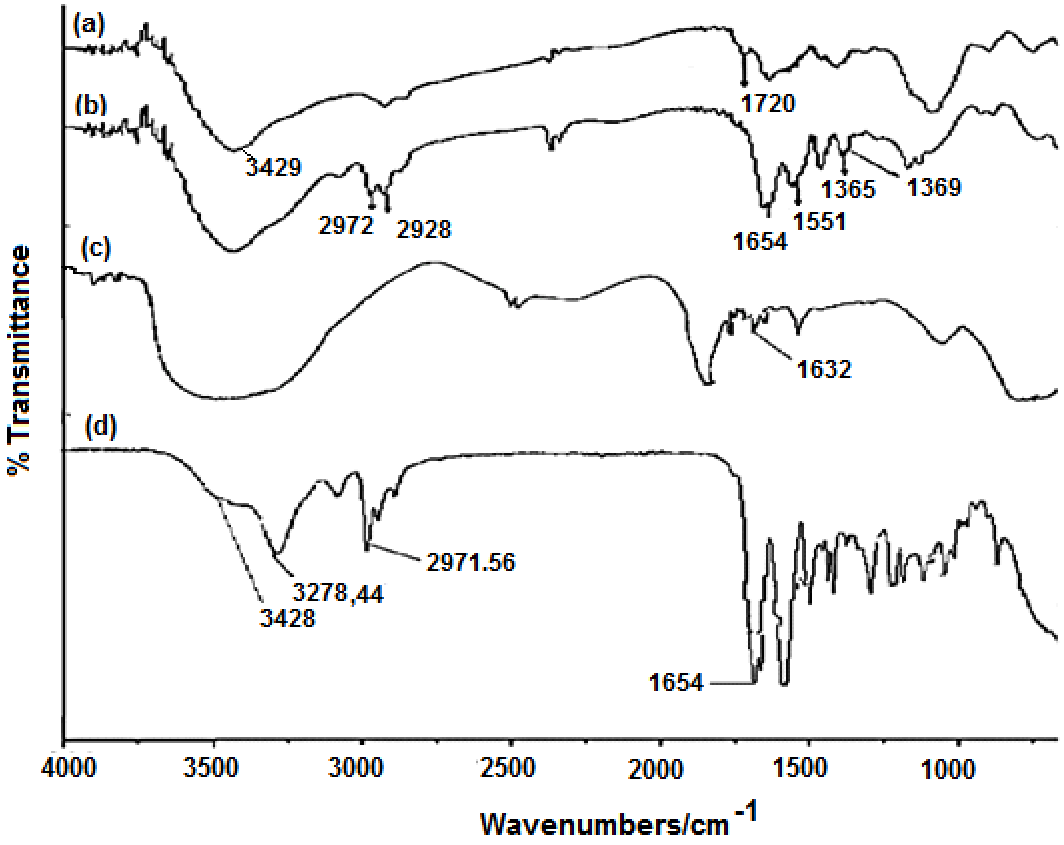

Figure S3: Vibrational-Spectroscopy-FTIR of Chitosan (a), PNIPAAm (b), PVP (c) and combinational CHT-PVP-PNIPAAm hydrogel (d).

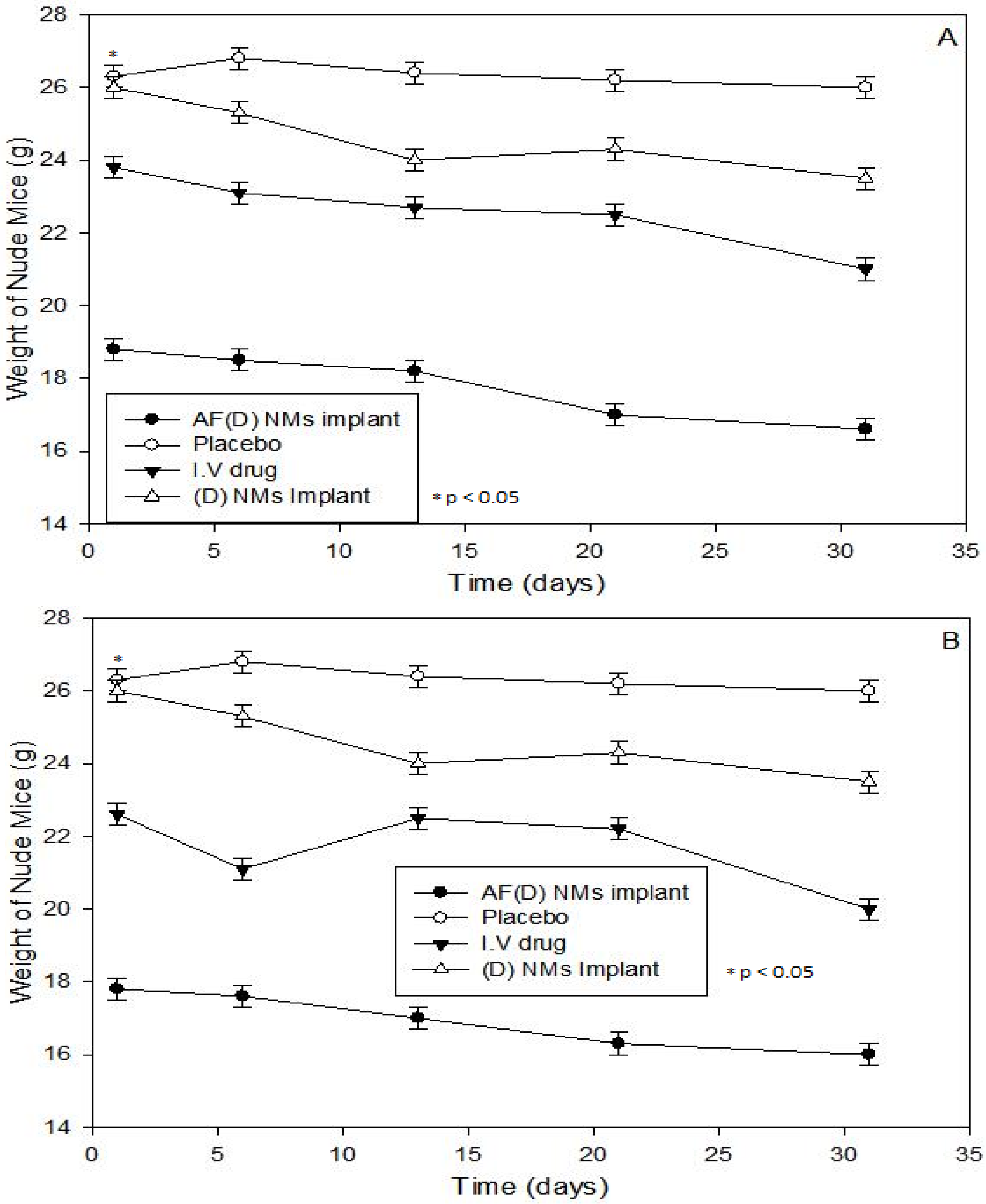

Figure S4. Nude mice average body weight curves illustrating chemotherapeutic efficacy in the 3 treatments and control (placebo) group. A refers to the methotrexate- and B the cisplatin-loaded PNIPAAm-b-PASP nanomicelle implant delivery system in NIH:OVCAR-5 EOC-bearing nude mice. Each point depicts mean (n=10/group); bar, ±SD. ***: $p < 0.05$ by t-test.**
